# Copy number variations in 119 Chinese children with idiopathic short stature identified by the custom genome-wide microarray

**DOI:** 10.1186/s13039-016-0225-0

**Published:** 2016-02-16

**Authors:** Guorui Hu, Yanjie Fan, Lili Wang, Ru-en Yao, Xiaodong Huang, Yiping Shen, Yongguo Yu, Xuefan Gu

**Affiliations:** Department of Pediatric Endocrinology and Genetic Metabolism, Xinhua Hospital, Shanghai Institute for Pediatric Research, Shanghai Jiao Tong University School of Medicine, 1665 Kongjiang Road, Shanghai, 200092 China; Medical Genetics Department, Shanghai Children’s Medical Center, Shanghai Jiao Tong University School of Medicine, Shanghai, 200127 China; Division of Endocrinology and Genetic Metabolism, Department of Internal Medicine, Shanghai Children’s Medical Center, Shanghai Jiao Tong University School of Medicine, Shanghai, 200127 China; Department of Laboratory Medicine, Boston Children’s Hospital, Boston, MA USA

**Keywords:** Idiopathic short stature, Custom chromosomal microarray, Copy number variation, East Asian population

## Abstract

**Background:**

Idiopathic short stature (ISS) refers to short stature with no evident etiologies. The custom genome-wide microarray specifically designed to cover height-related genes may be helpful to detect copy number variations (CNVs) in ISS patients, which may be missed by the general microarray. The aim of the study was to validate the applicability of the custom microarray and to analyze CNVs in Chinese ISS children.

**Results:**

Sixty non-polymorphic CNVs were identified in 119 ISS patients. There were 13 small CNVs with a size below 50 kb, accounting for 21.7 % of all the CNVs (13/60). Five pathogenic or possibly pathogenic CNVs were detected in five patients, including deletions at 22q11.21, duplications at 4q11-q13.1, 4q12 and Yp11.32-p11.2. Taking only the pathogenic variants into account, the diagnostic yield was 2.5 % (3/119). The *TMEM165*, *POLR2B* and *PDGFRA* genes were analyzed as candidate genes. A 15 kb deletion in the *RASA2* gene was of interest for further investigation.

**Conclusions:**

This study showed that the custom microarray is applicable to detect CNVs in patients with short stature. Candidate genes and CNVs detected in ISS patients may be helpful for CNV analysis of short stature, especially in East Asian population.

**Electronic supplementary material:**

The online version of this article (doi:10.1186/s13039-016-0225-0) contains supplementary material, which is available to authorized users.

## Background

Short stature is a common reason for referral to a pediatric endocrine clinic. Although a thorough diagnostic workup is performed including detailed medical history, physical examination, biochemical and radiologic screening, no definite cause can be established in approximately 80 % children [1]. As height is a classic polygenic trait, genetic testing consisting of target gene sequencing and chromosome karyotype is the next step if the clinician suspects a specific genetic disorder. However, a diagnosis of idiopathic short stature (ISS) is made in most cases in the end. ISS is defined as a condition in which the height of the individual is below 2 standard deviation score (SDS) of the corresponding mean height for a given age, sex and population group, and no known causes can be found such as intrauterine growth retardation, undernutrition, chronic pediatric illness, growth hormone deficiency, and other dysmorphic syndromes [2].

Chromosomal microarray (CMA), which has been recommended as the first-tier clinical diagnostic test for individuals with developmental disabilities or congenital anomalies [3], has a much higher resolution than conventional cytogenetic methods to screen genomic imbalances and can better define the size. The imbalances characterized by CMA are called copy number variations (CNVs). A CNV refers to an imbalance of a genomic sequence that alters the diploid status of a particular locus in the human genome. CNVs comprise deletions and duplications with DNA fragments ranging from 1 kilobase (kb) to several megabases (Mb) [4]. Rare CNVs, especially genomic deletions, were shown to be a contributor to short stature [5, 6]. Recent studies showed that pathogenic CNVs were found in 4–10 % of children referred for short stature, indicating that CNV assessment cannot be neglected for the evaluation of short stature without apparent diagnostic clues [6, 7]. Although the outcomes of these studies are promising, there are still some underlying problems. First, some small pathogenic imbalances may be missed. Although a CNV is defined to be as small as 1 kb, most studies excluded the CNVs less than 50 kb in consideration of the accuracy of diagnosis [3, 7, 8], as most general CMA does not have sufficient coverage to precisely call such small CNVs. Second, it is difficult to identify the actual genes responsible for short stature in large CNVs. In general, large CNVs are gene-rich, and several genes may be associated with growth. It is a great challenge to distinguish actual disease-causing genes among them with limited information. In small pathogenic CNVs with few genes, especially with only one, it will be easier to find a new gene associated with short stature for further study. The custom CMA designed with target genes may be a better solution. In recent years, some genome-wide association studies (GWAS) for common variants in large populations of individuals have been performed to discover genomic loci associated with height [9, 10]. More than 1077 candidate genes have been considered to associate with height [11]. However, the relationships between most of these genes and height have not been well established since there were few clinical reports.

In the present study, we firstly used the height-related candidate genes to design a genome-wide CGH + SNP microarray (CGH, comparative genomic hybridization; SNP, single nucleotide polymorphism), and then performed CNV detection in Chinese children with ISS in an effort to validate the applicability of this custom CMA as well as to reveal potential genes and loci related to short stature.

## Results

A total of 119 children from different families were included in the present study. The cohort exhibited a male predominance (61.3 %, 73/119). During the first evaluation, the chronological age was 8.6 ± 3.4 years. The mean SDS of length and weight at birth were −0.40 and −0.24, respectively, which were within the normal range. Postnatal growth restriction was obvious with the mean SDS of height below −2 (Table 1).

**Table 1:** Overview of the characteristics of the 119 patients

Feature	
Total, n	119
Male/Female, n (%)	73(61.3)/46(38.7)
Age at the first evaluation^a^ (years)	8.6 ± 3.4
SDS of length at birth^a^	−0.40 ± 1.02
SDS of weight at birth^a^	−0.24 ± 1.19
SDS of height at presentation^a^	−2.96 ± 0.97

*SDS* standard deviation score

^a^The value was shown as the mean ± standard deviation (SD)

Sxity non-polymorphic CNVs with the size ranging from 2 kb to 9 Mb were detected in 30 individuals after filtering the raw data with the DGV database. There were 13 small CNVs with the size below 50 kb, accounting for 21.7 % (13/60) of the CNVs identified. A total of five rare CNVs thought to be pathogenic or possibly pathogenic were detected in five patients, including two deletions and three duplications (Additional file 1: Figure S1).

In type I CNVs, deletions at 22q11.2 were found in two patients (patient 1 and 2) overlapping with the known deletion syndrome (MIM 611867). Patient 3 had a duplication containing partial *SHOX* gene that has been considered to associate with ISS (MIM 300582). Two type II CNVs were detected in patient 4 and 5 with a duplication located in 4q11-q13.1 and 4q12, respectively. Three genes, *TMEM165*, *POLR2B* and *PDGFRA*, were considered as the height-related candidate genes after searching supporting references. They were newly identified genes associated with human height in GWAS study and were all included in our target genes list. All the genetic findings were summarized in Table 2.

**Table 2:** Summary of type I and II CNVs

Patient	Karyotype^a^	Size (Mb)	No. of genes	DECIPHER^b^	Syndrome	Candidate gene
Type I						
1	arr 22q11.21(18729944–21704972) × 1	3	64	19	22q11.2	*TBX1*
2	arr 22q11.21(20659547–21704972) × 1	1	21	16	22q11.2	-
3	arr Yp11.32-p11.2(545173–10045809) × 3	9.5	50	11		*SHOX*
Type II						
4	arr 4q11-q13.1(52697788–59679060) × 3	7	39	0	-	*TMEM165, POLR2B*
5	arr 4q12(54749407–55157199) × 3	0.4	4	0	-	*PDGFRA*

^a^Genomic coordinates were based on Human Genome Building GCRh37, hg19

^b^Number of overlapping CNVs reported in the DECIPHER database with the feature of postnatal-onset short stature

Short stature was the only distinct symptom in the majority of the patients according to the medical records. As to the patients carrying type I or II CNVs, delayed bone age was common. Other available clinical information was shown in Table 3.

**Table 3:** Clinical information of patients with type I and II CNVs

Patient	Gender	Age (years)	Height (cm), SDS	Weight (kg)	Clinical findings
Type I					
1	M	NA	NA	NA	NA
2	M	7.8	110.8, −3.4	15.5	BA 4 years, BMI 12.6, normal heights of parents
3	M	7.3	115.3, −2.0	25.2	BA 6 years, BMI 18.9, normal heights of parents
Type II					
4	M	16.3	152.8, −3.1	43	BA 13 years, BMI 18.4, multiple pigmented nevi on the face, normal heights of parents
5	M	11.3	128, −2.8	21	BA 6 years, BMI 12.8, ptosis, normal heights of parents

*M* male, *SDS* standard deviation score, *BA* bone age, *BMI* body mass index, *NA* not available

Only type III and IV CNVs or polymorphic CNVs were found in the other 114 patients. As the CMA testing of most parents and control group was not available, type III and IV CNVs could not be clearly distinguished. At last, all the CNVs categorized as benign or uncertain significance were summarized in Additional file 1: Table S2. A 15 kb deletion in the *RASA2* gene was of potential interest although it was classified into type III (Additional file 1: Figure S1).

Taking only the pathogenic variants into account, the overall diagnostic yield of CMA testing for patients with ISS was 2.5 % (3/119).

## Discussion

Short stature is a common symptom in a variety of chromosome imbalances and monogenic disorders [12]. It is a challenge for clinicians to make a precise diagnosis relying on a general diagnostic workup such as standard physical examination and laboratory parameters that assess the function of growth hormone axis. With tremendous advance of techniques in genetic diagnostics and understanding of the genetic basis of growth, genetic evaluation has played a growing role in elucidating the underlying causes of growth disorders. If a child is diagnosed with ISS, CMA testing and next-generation sequencing are recommended [13].

So far, there were only four studies in which CMA testing was used to identify pathogenic CNVs in children with short stature [6–8, 14]. Two of them involved in children either born with normal birth size or born with small for gestational age (SGA), and the other two concerned the children born only with SGA. With the general CMA, at least one disease-causing CNV was detected in 4–10 % of patients without considering the birth size and weight [13] or in ~16 % of patients born with SGA [8, 14]. More studies are needed to explore genetic etiologies on the chromosome level of unexplained short stature. In the present study, we performed CMA testing in 119 patients with ISS using a custom microarray. As a result, non-polymorphic CNVs with a size as small as a few kb could be detected by our microarray. Among them, small CNVs with a size below 50 kb accounted for 21.7 %, suggesting that small CNVs could not be ignored for study. As these small CNVs were not included in pathogenic or possibly pathogenic group, they were not further validated by qPCR. The log2 ratio used for CNV calling was very strict in the study, which was produced from the practical measure of Agilent Company, thus most of the small CNVs were likely to be true.

In the present study, the size of CNVs thought to be pathogenic or possibly pathogenic was more than 400 kb with a diagnostic yield of 2.5 % among children with ISS. The frequency was low compared with previous reports. This outcome was reasonable. Analyzing the previous four studies, we found that pathogenic CNVs seemed to be more common in patients born with SGA than in patients born with normal length and weight. In addition, the diagnostic yield in the present study was calculated taking only the pathogenic variants (type I CNVs) into account. All the pathogenic CNVs were assessed based on several supporting literatures, indicating that the pathogenicity of these CNVs was widely received. Therefore, our results demonstrated the applicability of the custom CMA to detect CNVs, especially those with small size. For instance, a 15 kb intragenic deletion in the *RASA2* gene, which was included in our target genes list, was detected and was validated to be true. In a previous study [6], rare CNVs were further filtered with control group after excluding polymorphic CNVs in the DGV database, and all the CNVs smaller than 50 kb were not retained. While in our study, 13 CNVs with a size below 50 kb were retained after exclusion of polymorphic variants in the DGV database, indicating that a further filtration with control group was necessary. Large control group may be needed for analyzing the pathogenicity of CNVs detected in East Asian population. Although many CNVs in the study cannot establish the association with short stature at present, several identified CNVs, especially some small ones, may be helpful for future studies.

The 22p11.21 deletions were found in two patients (patient 1 and 2). These interstitial deletions were part of the chromosome 22q11.2 deletion syndrome (MIM 611867, chr22:17,900,000-25,900,000, hg19). This deletion syndrome was very common with an estimated frequency of 1:2000–1:4000 [15]. There were many possible signs and symptoms that might affect almost any aspect of the body. Growth restriction was often found when a deletion was involved this region [7, 8, 14, 16, 17]. The 22q11.2 microdeletions varied in size between ~700 kb and ~3.6 Mb [16, 18]. According to the new proposed categorization system [16], the region of 22q11.2 could be divided into eight critical components named LCR22-A to H (LCR, low copy repeat). The region of LCR22-A to –D was assigned as the proximal 22q11.2 region with ~3 Mb in size and the distal 22q11.2 region comprised LCR22-D to –H. Deletion of the proximal region presented the clinical manifestations of the recurrent DiGeorge/Velocardiofacial syndrome (DGS/VCFS) (MIM 188400 and 192430). Haploinsufficiency of the *TBX1* gene in particular was responsible for most of the physical malformations of these two syndromes. In our study, the ~3.0 Mb deletion in patient 1 was the typical DGS/VCFS region containing the *TBX1* gene. Unfortunately, the outpatient medical records of the patient were lost and no more clinical information could be reviewed. In patient 2, the 22q11.21 deletion was located in the proximal 22q11.2 region spanning LCR-B to –D in which the *TBX1* gene was not included. Growth restriction was the only clinical phenotype in patient 2. It has been reported in previous study that similar growth restriction was the only phenotype [7].

A 9.5 Mb duplication containing part of the *SHOX* gene in chromosome Y was detected in patient 3. One copy of the *SHOX* gene is located on each of the sex chromosomes (the X and Y chromosomes) in an area called the pseudoautosomal region. Although many genes are unique to either the X or Y chromosome, genes in the pseudoautosomal region are present on both chromosomes. As a result, both females (who have two X chromosomes) and males (who have one X and one Y chromosome) have two functional copies of the *SHOX* gene in each cell. The protein encoded by this gene is a transcription factor and plays a particularly important role in the growth and maturation of bones in the arms and legs. Deletion or duplication of the *SHOX* gene is a well-established cause of ISS [19] (MIM 300582). Moreover, partial *SHOX* duplications appeared to have a more deleterious effect on skeletal dysplasia and height gain than complete *SHOX* duplications [19].

In patient 4, a 4q11-q13.1 duplication was detected, while the other duplication of a smaller region at the same position (4q12) was identified in patient 5. The pathogenicity of the large duplication in patient 4 was evaluated based on the size of the CNV as well as two genes, *TMEM165* and *POLR2B*. Mutations in the *TMEM165* gene cause congenital disorder of glycosylation, type IIk in which postnatal growth retardation is a common symptom [20]. In the DGV database, there were not any reports of duplications or large deletions in the region of the gene, implying the rareness of CNVs comprising the *TMEM165* gene. The *POLR2B* gene encodes the second largest subunit of RNA polymerase II (Pol II), a DNA-dependent RNA polymerase that catalyzes the transcription of DNA into precursors of mRNA, snRNA and microRNA. It has been shown to strongly associate with human height in the GWAS study [10]. Biological pathways analysis showed that POLR2B protein could interact with OBSL1 protein, which was associated with one of the commonest primordial growth disorders, 3-M syndrome [21]. Without further information, both *TMEM165* and *POLR2B* were deemed as candidate genes in patient 4. In patient 5, the *PDGFRA* gene was one of the 84 genes related to the JAK/STAT pathway through which growth hormone promotes cellular growth and proliferation [22]. Moreover, low *PDGFRA* expression negatively affected the body size not only in the mice but also in human [23]. This gene encodes platelet-derived growth factor receptor alpha which plays a role in the development of bone [24, 25]. We suspected that partial duplication of the *PDGFRA* gene in patient 5 disrupted the expression of this gene and contributed to ISS.

In the group of uncertain significance, a 15 kb microdeletion found in a seven-year-old boy was of interest. The heterozygous deletion spanned only the exon 6 to 9 of one gene, the *RASA2* gene. The protein encoded by this gene is a member of the GAP1 family of GTPase-activating proteins. Acting as a suppressor of RAS function, the protein enhances the weak intrinsic GTPase activity of RAS proteins resulting in the inactive GDP-bound form of RAS, thereby allowing control of cellular proliferation and differentiation. Causative mutations in several genes encoding components of the RAS/MAPK ERK pathway have been demonstrated to result in short stature, and the group of disorders is termed RASopathies [26]. Noonan syndrome (NS) is one of the RASopathies. Peng-Chieh Chen et al*.* [27] reported that three patients of NS carried a different heterozygous mutation in *RASA2* gene, respectively. Moreover, expression of the mutant *RASA2* alleles in heterologous cells increased RAS-ERK pathway activation, supporting a causative role in NS pathogenesis. As to our patient, he only presented hyperkeratosis of skin as well as short stature, which was corresponding to the features of NS. However, this CNV was validated to derive from his mother who was normal. As a result, it was classified into VUS.

## Conclusions

In conclusion, in the present study, we detected five rare CNVs as the potential causes of ISS in 119 children. The applicability of the custom genome-wide microarray was validated to detect CNVs in patients with short stature, with a diagnostic yield of 2.5 %. Several candidate genes were identified to be associated with growth based on the bioinformatics analyses, and further studies were necessary to clarify their involvement in the growth pathways. A number of CNVs, which were classified to be benign or VUS, might be useful for further study to analyze the pathogenicity of CNVs detected in patients with short stature. Our results expanded the potentially genetic causes and candidate genes of ISS in East Asian population.

## Methods

### Patients and ethics

Patients were selected from the children referred to our pediatric endocrine clinic due to short stature using the following criteria [28]: (1) short stature with height SDS ≤ −2; (2) no obviously abnormal body proportions; (3) normal length and weight for gestational age at birth; (4) normal food intake; (5) no evidence of known causes of short stature [2], including endocrine diseases (growth hormone deficiency and hypothyroidism), skeletal dysplasias and systemic diseases such as Turner syndrome.

The study was approved by the by the ethics committee of Xinhua Hospital and informed consent form was obtained from all families.

### Chromosome microarray design and testing

The array platform was designed as a 4 × 180 K CGH + SNP microarray by Agilent Technologies Inc. (Santa Clara, CA, USA). This array contained ~110,000 oligonucleotide probes for the detection of CNVs, and ~60,000 SNP probes for the detection of loss of heterozygosity (LOH) as well as CNVs. It contained high-density coverage for 1469 target genes (Additional file 1: Table S1) with an average oligo probe spacing of ~5 kb or at least 20 probes per gene. These target genes were summarized from previous studies [11] and some unpublished data of our own study. In addition, genome-wide backbone coverage was included with an average probe spacing of ~25 kb.

Genomic DNA was extracted from peripheral blood leucocytes of all patients using the standard procedure of the lab. The CMA testing was conducted according to the manufacturer’s protocol. The results were processed using the Agilent CytoGenomics Edition 2.9.2.4 software (Agilent Technologies, Inc.). The adjusted Aberration Detection Method 2 analysis algorithm was applied for CNV calling with a sensitivity threshold of 6.0. Briefly, at least 3 continuous probes were required to call an aberration of a genomic segment as a duplication or a deletion. The log2 ratio of test/reference intensities >0.53 was considered duplication, whereas the ratio <−0.9 was considered deletion. All the coordinates of CNVs were based on NCBI human genome build 37 (hg19).

### CNVs analysis and validation

All detected CNVs were firstly compared with data in the Database of Genomic Variants (DGV) [29] to exclude polymorphic CNVs. The remaining CNVs were preliminarily evaluated for pathogenicity considering the following conditions [30]: (1) overlap with genomic coordinates for a known genomic imbalance syndrome; (2) gene content of coding genes associated with impaired developmental process and/or impaired cell growth pathways and/or growth impairment in animal models; (3) more than 3 Mb in size, especially for microdeletion; (4) overlap with CNVs defined in other patients presenting postnatal short stature reported in the Database of Chromosomal Imbalance and Phenotype in Humans using Ensembl Resources (DECIPHER) [31]. As a result, CNVs were classified into four categories: (I) known pathogenic CNVs (known microduplication or microdeletion syndromes); (II) possibly pathogenic CNVs in which there were genes with evidence supporting the pathogenicity; (III) variants of uncertain significance (VUS) if they were unable to be classified in one of the two categories mentioned above; (IV) probably benign if the CNV was identified in several patients without pathogenic evidence or there was no gene in the interval. A CNV only involved in non-coding areas was also thought to be benign.

All type III and IV CNVs were not further assessed. Type I and II CNVs were validated twice with two pairs of primers in different genes (Additional file 1: Table S3) by quantitative real-time polymerase chain reaction (qPCR) using the SYBR Premix Ex Taq (Tli RNaseH Plus) kit (Takara Bio Inc., Terra Bella, CA, USA) on a ViiA7 or StepOnePlus Real-Time PCR System (Life Technologies, Carlsbad, CA, USA). If the genomic DNA of the patient’s parents was available, the occurrence (de novo or inherited) status would be provided.

All validated type I and II CNVs were further evaluated. Gene function was collected from the Online Mendelian Inheritance in Man (OMIM) and NCBI Entrez Gene databases. The information regarding gene involved in growth impairment in mouse model was collected from the murine knockout phenotypes in the Mouse Genome Informatics (MGI) database [32]. The function of microRNAs in the CNVs was evaluated using the miRBase and miRTarBase databases [33, 34]. PubMed and Google Scholar databases were searched to obtain as much information as possible.

## Additional file

Additional file 1: Table S1. List of 1469 height-associated candidate genes analyzed by genome-wide association studies. **Table S2.** Summary of type III and IV CNVs. **Table S3.** Primers of qPCR. **Figure S1.** Screenshots of chromosome microarray and qPCR. (DOC 7216 kb)

## Abbreviations

CMA: chromosomal microarray
CNV: copy number variation
DECIPHER: Database of Chromosomal Imbalance and Phenotype in Humans using Ensembl Resources
DGV: Database of Genomic Variants
GWAS: genome-wide association study
ISS: idiopathic short stature
kb: kilobase
Mb: megabase
NS: Noonan syndrome
OMIM: Online Mendelian Inheritance in Man
SDS: standard deviation score
SGA: small for gestational age
VUS: variant of uncertain significance
