# Supplementary material for: Copy number variations in 119 Chinese children with idiopathic short stature identified by the custom genome-wide microarray
Source: Mol Cytogenet. 2016 Feb 16;9:16. doi: 10.1186/s13039-016-0225-0 (PMC4755006; doi:10.1186/s13039-016-0225-0)
Supplement: Additional file 1: Table S1. — List of 1469 height-associated candidate genes analyzed by genome-wide association studies. Table S2. Summary of type III and IV CNVs. Table S3. Primers of qPCR. Figure S1. Screenshots of chromosome microarray and qPCR. (DOC 7216 kb) [file 13039_2016_225_MOESM1_ESM.doc]

**Copy number variations in 119 Chinese children with idiopathic short stature identified by genome-wide custom microarray**

Table S1 List of 1469 height-associated candidate genes analyzed by genome-wide association studies

Table S2 Summary of type III and IV CNVs

Table S3 Primers of qPCR

Fig. S1 Screenshots of chromosome microarray and qPCR. From A to F, they were patient 1 to 5 and a boy categorized into variants of uncertain significance. N1 to 3 were normal controls.

Table S1 List of 1469 height-associated candidate genes analyzed by genome-wide association studies

ABAT

ABCA13

ABCB5

ABCC4

ABCC8

ABCE1

ABHD12

ABP1

ACACA

ACCS

ACPL2

ACSF3

ACTN1

ACTR3

ACVR1

ACVR1B

ADA

ADAM12

ADAM28

ADAMTS10

ADAMTS12

ADAMTS16

ADAMTS17

ADAMTS2

ADAMTS20

ADAMTS3

ADAMTSL3

ADCY2

ADCY3

ADCY4

ADH5

ADIPOR2

ADRA1A

ADRBK1

AEBP2

AF068195

AF085837

AF111178

AF315099

AGAP1

AGPAT4

AGPS

AGRN

AHR

AIFM1

AJ420566

AK024373

AK025394

AK054997

AK056769

AK094352

AK130123

AK130913

AK291370

AK296158

AK297601

AK302380

AK304777

AK309891

AK311874

AK5

AK8

AKAP6

AKD1

AKT3

AL133110

AL360139

ALDH1A3

ALG12

ALK

ALMS1

ALPL

ALPP

ALS2

ALX4

AMBRA1

ANKH

ANKRD13D

ANKRD17

ANKRD26

ANKRD6

ANKS1A

ANKS1B

ANO5

ANO7

ANTXR1

ANTXR2

AP3D1

ARFGEF2

ARHGAP15

ARHGAP18

ARHGAP24

ARHGAP5

ARHGAP6

ARHGEF18

ARHGEF3

ARHGEF7

ARID1B

ARL6

ARPC1A

ARPC2

ARSB

ASB12

ASS1

ASTN1

ASTN2

ATAD2B

ATF2

ATG7

ATG9B

ATP11B

ATP13A2

ATP2B1

ATP2B2

ATP6V0A2

ATP7A

ATP8B1

ATP9B

ATR

ATRX

ATXN3

AXDND1

AXIN2

B3GALTL

B3GNTL1

B4GALNT3

B4GALT1

BACH2

BAI3

BANK1

BARX2

BBS1

BBS12

BBS2

BBS4

BBS5

BBS7

BBS9

BBX

BC003593

BC014370

BC015798

BC040589

BC062672

BC141544

BC142620

BC146488

BC160127

BC171825

BCAS3

BCKDHA

BCKDHB

BCL2

BCL2L14

BCL7A

BCL9

BCOR

BCR

BFSP2

BICC1

BICD2

BIN1

BMP1

BMP2

BMP2K

BMP5

BMP6

BMP7

BMPR1A

BMPR1B

BMPR2

BNC2

BPIL2

BRAF

BRCA2

BTBD9

BTC

BTN1A1

BUB1B

BX538158

C13orf1

C13orf18

C14orf118

C14orf145

C14orf149

C14orf39

C15orf23

C17orf37

C18orf45

C1orf21

C1orf87

C20orf152

C20orf191

C20orf26

C20orf4

C21orf56

C2orf78

C2orf84

C3orf32

C3orf62

C3orf63

C3P1

C4orf31

C4orf37

C6orf1

C6orf106

C6orf125

C6orf138

C6orf142

C6orf191

C6orf97

C7orf41

C7orf50

C8orf12

C8orf74

C8orf80

C9orf40

C9orf41

C9orf93

CA1

CA2

CA8

CABLES1

CACNA2D1

CACNA2D2

CACNA2D3

CACNA2D4

CADM1

CADM2

CADPS

CADPS2

CAGE1

CALCRL

CALN1

CAMK1D

CAMKMT

CANT1

CAP2

CARD11

CARKD

CASK

CASKIN1

CASR

CAST

CATSPERB

CAV3

CCBL2

CCDC102B

CCDC3

CCDC50

CCDC66

CCDC77

CCDC85A

CCDC88A

CCDC91

CCNB2

CCNY

CCRN4L

CD2AP

CD38

CD84

CDC14B

CDC16

CDC42BPG

CDCA2

CDCA7

CDH10

CDH15

CDH3

CDON

CELF5

CENPC1

CENPI

CENPP

CEP120

CEP164

CEP192

CEP290

CERKL

CHAF1A

CHCHD3

CHD1L

CHD7

CHIC2

CHL1

CHN1

CHRNG

CKAP2

CLCN3

CLCN5

CLDN10

CLINT1

CLNK

CLOCK

CLU

CLUL1

CLYBL

CNOT6

CNPY1

CNTN4

CNTN5

CNTN6

CNTNAP2

COBLL1

COL11A1

COL11A2

COL1A1

COL3A1

COL4A1

COL4A2

COL5A1

COL5A2

COL6A2

COL6A5

COL9A1

COPA

COX18

CPAMD8

CPEB2

CPEB3

CPEB4

CPN1

CPNE8

CPNE9

CR603564

CR616400

CR620263

CRADD

CREB5

CREBBP

CRHR1

CRMP1

CROCC

CRTAP

CSE1L

CSN3

CSNK1G1

CSNK1G3

CTAGE5

CTDP1

CTIF

CTNNA1

CTNNA2

CTNNA3

CTNND2

CTPS

CTSK

CXCR1

CXXC4

CYB5A

CYP19A1

CYP27A1

CYP27B1

DAAM1

DAB1

DACT1

DCHS2

DCLRE1C

DDHD1

DDX21

DDX27

DDX4

DDX50

DDX6

DEAF1

DENND1B

DEPTOR

DFFB

DGKZ

DHCR24

DHDDS

DIAPH2

DIAPH3

DIP2A

DIP2C

DIRC2

DIS3L2

DKFZp686K1684

DLAT

DLEU7

DLG2

DLG5

DLGAP2

DLL1

DLL3

DMD

DNAH5

DNAJC15

DNM3

DNMBP

DNMT3A

DOCK1

DOCK2

DOCK3

DOCK5

DOK6

DOT1L

DPCD

DPYSL2

DQ451147

DSCAM

DTL

DUSP16

DYM

DYRK1A

E4F1

EBF2

ECHDC1

EEFSEC

EEPD1

EFCAB11

EFEMP1

EFHD1

EFNB2

EFR3B

EFTUD2

EGR3

EIF2AK2

EIF2AK3

EIF4B

EIF4E3

ELFN2

ELK4

ELMOD3

EMBP1

EMID2

ENOX1

ENOX2

ENPP2

EP300

EPB41L1

EPB41L2

EPC1

EPHA6

EPHB2

EPHX2

EPRS

EPYC

ERBB2IP

ERBB4

ERC1

ERC2

ERCC2

ERCC3

ERI3

ERLIN1

ESCO2

ESR1

ESR2

ETNK1

ETS1

ETV6

EU250754

EVC

EVC2

EXOC2

EXOC4

EXT1

EXT2

EYS

FAM114A2

FAM124B

FAM172A

FAM184B

FAM190A

FAM19A1

FAM19A2

FAM19A4

FAM21C

FAM46A

FAM49B

FAM65B

FAM70A

FAM78B

FAM82A1

FANCA

FANCC

FANCD2

FANCI

FANCL

FANCM

FARP1

FARP2

FASTKD3

FAT1

FAT4

FBF1

FBLN1

FBLN2

FBLN5

FBN1

FBN2

FBXL17

FBXL7

FBXO15

FBXO7

FBXW11

FCHO2

FEM1A

FER

FGD4

FGF1

FGF10

FGF12

FGF13

FGF14

FGF18

FGF23

FGFR1

FGFR1OP

FGFR2

FGFR3

FGFR4

FHIT

FILIP1

FIP1L1

FJ423745

FKTN

FLI1

FLJ22536

FLJ39739

FLJ41941

FLNB

FMO4

FMO5

FMR1NB

FNDC1

FNDC3A

FNDC3B

FNIP1

FOSL1

FREM3

FRMD4A

FRMPD4

FRS2

FRZB

FSD1L

FSHR

FTCD

FUCA1

FXR2

FYB

GAB1

GABRA1

GABRA3

GABRA6

GALNS

GALNTL6

GAP43

GATA4

GATM

GCC2

GCNT2

GDNF

GDPD5

GFM1

GFPT2

GHR

GHRHR

GIPC2

GJC3

GK

GKAP1

GLB1

GLDC

GLI2

GLI3

GLIS3

GLRA1

GLRA2

GLRA3

GLT25D2

GMPR2

GNA12

GNAS

GNN

GNPAT

GNPTAB

GNRHR

GPC3

GPC4

GPC5

GPC6

GPD1L

GPHN

GPM6A

GPR115

GPR126

GPR143

GPR39

GPR98

GPSM1

GRB10

GRB14

GRIA3

GRID2

GRIK1

GRK7

GRM4

GRM7

GRM8

GRPEL2

GRTP1

GRXCR2

GSR

GTDC1

GTF2E2

GULP1

H2AFY

HABP4

HACE1

HAGH

HAX1

HDGFRP3

HDLBP

HEMK1

HEPACAM2

HEXIM1

HIBCH

HIF1A

HINT3

HIRA

HLA-DQA1

HLA-DQA2

HLA-DRB5

HLF

HMBOX1

HMGA2

HMGN2P46

HNRNPF

HOXA11

HOXD3

HPRT1

HPSE

HPSE2

HQ448490

HS2ST1

HS6ST2

HS6ST3

HSPG2

HTR2C

HTT

HUNK

ICK

IFT57

IFT80

IGBP1

IGF1

IGF1R

IGF2BP2

IGF2BP3

IGF2R

IGFBP2

IGFBP7

IGSF22

IKBKAP

IL17RC

IL17RD

IL1RAP

IL1RAPL2

IL31RA

IMMP1L

IMMP2L

IMPACT

INSR

INTS6

INTS7

INTS9

IPO7

IPPK

IQSEC3

IRAK2

IRF1

IRF2

IRS2

ITGA11

ITGA4

ITGA5

ITGAV

ITGB5

ITGB8

ITIH4

ITPR1

ITPR3

IWS1

JAG1

JAK2

JAKMIP1

JAZF1

JF432188

JF432216

KAL1

KCNH2

KCNIP4

KCNN3

KCNQ1

KCTD1

KDM4C

KDM5A

KDM5C

KDR

KHDRBS3

KIAA0317

KIAA0368

KIAA0430

KIAA0562

KIAA0586

KIAA0947

KIAA1211

KIAA1244

KIAA1267

KIAA1279

KIAA1468

KIAA1715

KIAA1751

KIAA1967

KIF13B

KIF1A

KIF23

KIF27

KIT

KLHL32

KLKB1

KRAS

KRT75

KRT84

KSR1

KYNU

L3MBTL3

LAMA2

LAMB4

LAMC1

LAMP1

LARGE

LARP4B

LASS3

LASS6

LBR

LCORL

LEMD3

LEPRE1

LETMD1

LHFPL4

LHX3

LHX4

LIAS

LIG4

LIN28A

LIN28B

LMBR1

LMCD1

LMO4

LMX1B

LNX1

LOC100507178

LOC144742

LOC285456

LOC339524

LOC375190

LOC553103

LOC643714

LOC645166

LONP2

LPA

LPAR1

LPGAT1

LPHN2

LPHN3

LPPR5

LRBA

LRIG3

LRP5

LRP6

LRRC15

LRRC16A

LRRC20

LRRC37A3

LRRC37B

LRRC47

LRRC57

LRRK1

LSAMP

LSS

LTBP1

LTBP2

LTK

LUZP1

LY86

LY86-AS1

LYPLAL1

MACF1

MACROD2

MAD1L1

MAGI1

MAGI2

MAK

MAMDC2

MAML2

MAP2K1

MAP2K3

MAP2K6

MAP3K4

MAP6

MAPK1

MAPK10

MAPK9

MAPT

MAST4

MASTL

MB21D2

MBD5

MBNL2

MBOAT1

MBP

MBTD1

MCM3AP

MCPH1

MCTP2

MDGA2

MECP2

MED12L

MED30

MEF2A

MEF2C

MEGF6

MESP2

MEST

METAP1D

METTL21D

METTL22

MFN2

MGAT5

MICAL3

MID1

MIPOL1

MIR548A2

MIR548F1

MIR548G

MIR548N

MIR548W

MIS12

MKL2

MLC1

MLF1

MLLT3

MLLT4

MLXIP

MMAA

MMP2

MMP24

MPHOSPH9

MSL2

MSRA

MTDH

MTHFD2L

MTM1

MTMR8

MTMR9

MTNR1A

MUSK

MUTED-TXNDC5

MYH11

MYH7B

MYO16

MYO18B

MYO1B

MYO1D

MYO1E

MYO1F

MYO6

MYO9A

MYO9B

MYT1L

NA

NAA35

NAB1

NAIP

NALCN

NASP

NBN

NCAPG

NCK1

NCKAP1

NCOA1

NCOA3

NCOA6

NCOR2

NDUFB5

NEDD8-MDP1

NEK3

NEK4

NELL2

NETO1

NF1

NFATC1

NFATC4

NFIC

NHS

NINJ2

NIPAL2

NIPBL

NKAIN2

NKAIN3

NLGN4X

NLRP7

NMBR

NME1-NME2

NMNAT2

NOC2L

NOS3

NOTCH2

NOVA1

NOX3

NPFFR2

NPHP3-ACAD11

NPR3

NR0B1

NR2E3

NRG1

NRG3

NRIP3

NSD1

NSDHL

NSF

NT5DC1

NTM

NTNG1

NTS

NUCB2

NUDT3

NUP160

NUP35

NUSAP1

OBSL1

OCRL

ODZ1

ODZ2

OLA1

OPN5

OPRM1

ORAOV1

ORC6

OSBPL11

OSBPL1A

OSBPL6

OSTF1

OSTN

OTUD4

OXSR1

PACRG

PACSIN1

PADI2

PAIP2B

PAK1

PALLD

PANK3

PAPPA

PAPPA2

PAPSS2

PAQR5

PARD3

PARK2

PARN

PARVA

PASD1

PAX3

PAX8

PCBD2

PCCB

PCDH9

PCGF3

PCLO

PCNT

PCSK5

PCYT1B

PDCL2

PDE10A

PDE11A

PDE1A

PDE3A

PDGFB

PDGFRA

PDIA4

PDK3

PDLIM4

PDS5A

PES1

PEX1

PEX5L

PEX7

PFAS

PGD

PHEX

PHF20

PHF6

PHLDB1

PIBF1

PIGK

PIGN

PIK3C2A

PITPNM2

PITX1

PJA2

PKN2

PLCE1

PLD1

PLD5

PLEKHA5

PLG

PLOD2

PLXNA1

PLXNA4

PLXNC1

PMEPA1

PML

PMM2

PMPCA

PNKD

PNOC

PNPT1

POLR2B

POLR3A

POLR3G

POPDC2

POR

POU1F1

PPA2

PPAP2A

PPAPDC1A

PPM1A

PPP1CC

PPP1R1C

PPP2R2A

PPP2R2C

PPP2R3A

PPP2R5A

PPP3CC

PQLC1

PRAM1

PRDM6

PREPL

PRICKLE1

PRIM2

PRKAB2

PRKAR1A

PRKCA

PRKCE

PRKCZ

PRKG1

PRKG2

PRKX

PRKY

PRMT2

PROK2

PROM1

PRPF8

PRR5

PRR5-ARHGAP8

PRRC1

PSMD12

PSORS1C1

PTAR1

PTCH1

PTCH2

PTCHD1

PTEN

PTGFR

PTGS2

PTH

PTH1R

PTK2B

PTPN11

PTPRD

PTPRE

PTPRJ

PTS

PTTG1IP

PVRL1

PXDN

PXK

PYGB

PYGO1

QKI

QSOX2

RAB1A

RAB27A

RAB3GAP1

RAB3GAP2

RAD18

RAD23B

RAD51L1

RALBP1

RALYL

RAP1GDS1

RAPGEF4

RAPGEF6

RASA2

RASGEF1B

RASGRP1

RASGRP3

RB1

RBBP8

RBFOX1

RBFOX2

RBM22

RBM28

RBM33

RBMS3

RCBTB1

RCHY1

RCSD1

RFC1

RFC3

RFK

RFT1

RFWD2

RFX6

RGPD1

RGS12

RHOA

RIOK2

RIPK3

RNASET2

RNF135

RNF139

RNF24

ROBO1

ROR2

RORA

RPA1

RPAP2

RPL22L1

RPS6KA2

RPS6KA3

RREB1

RRN3

RSPO3

RTF1

RTN4RL1

RUFY3

RUNX2

RUNX3

RYBP

RYR2

RYR3

S100B

SALL1

SALL3

SALL4

SAP130

SBNO1

SBNO2

SCAP

SCARB2

SCARF1

SCG5

SCMH1

SCN9A

SCUBE2

SCUBE3

SDHA

SDHB

SEC16A

SEMA3A

SEMA3E

SEMA5A

SENP6

SERPINE2

SERPINF2

SERTAD2

SETD5

SF3B3

SFMBT1

SGCZ

SH3BP2

SH3GL3

SHH

SHOX2

SHQ1

SHROOM2

SHROOM4

SIL1

SIRT4

SIX3

SKAP2

SKI

SLC12A6

SLC12A7

SLC15A1

SLC16A7

SLC16A9

SLC22A1

SLC22A2

SLC22A3

SLC22A4

SLC22A5

SLC25A14

SLC34A1

SLC35D1

SLC35D2

SLC35F5

SLC38A9

SLC39A14

SLC40A1

SLC43A2

SLC4A10

SLC4A4

SLC5A7

SLC6A1

SLC6A11

SLC6A12

SLC6A13

SLC6A6

SLC6A8

SLC8A1

SLCO1A2

SLCO1B3

SLIT2

SLIT3

SLMAP

SMAD1

SMAD5

SMAD9

SMARCA5

SMARCAL1

SMC1A

SMCHD1

SMEK2

SMO

SMS

SMYD3

SMYD4

SNAP47

SNCA

SNED1

SNTG1

SNTG2

SNX18

SOCS7

SOD2

SOS1

SOX5

SOX6

SPAG16

SPAG9

SPATA18

SPIRE2

SPP1

SPRED2

SPTB

SRBD1

SRD5A1

SRGAP3

SRGN

SRPK2

SSFA2

SSR1

ST3GAL1

ST7

ST7L

ST8SIA4

ST8SIA6

STAG1

STAP1

STARD3NL

STAT1

STAT3

STAT4

STAT5A

STC2

STK10

STK11

STK24

STK25

STK32B

STL

STON1-GTF2A1L

STXBP5L

SUCLG2

SUGP2

SULT1E1

SUMF1

SUPT3H

SUZ12

SYN2

SYN3

SYNE1

SYNJ1

SYTL4

TACR3

TAF2

TAOK1

TAZ

TBC1D21

TBL1X

TBL1XR1

TBL1Y

TBP

TBPL2

TBX1

TBX15

TC2N

TCF25

TCF4

TCF7L2

TCOF1

TDP1

TDRD5

TEAD1

TEP1

TERT

TET2

TFDP2

TGFB1

TGFB2

TGFBR1

TGFBR2

TGFBRAP1

THOC5

THRA

THRB

THSD4

THSD7B

TIAM1

TIAM2

TINAG

TLE3

TLN2

TMEM165

TMEM176A

TMEM181

TMEM185A

TMEM192

TMEM232

TMEM30A

TMEM38B

TMF1

TMPRSS11D

TMPRSS6

TNC

TNFAIP8

TNFRSF10B

TNFRSF10D

TNFRSF11B

TNFRSF8

TNFSF13B

TNMD

TNPO1

TNS1

TOP3A

TOX

TOX3

TP53I13

TP63

TP73

TPD52L1

TPO

TRA2B

TRAF7

TRAPPC2

TRIM37

TRIP11

TRMT11

TRMT2B

TRPM5

TRPS1

TRPV4

TSC22D2

TSNAX-DISC1

TSPAN5

TTC27

TTC7A

TTK

TTLL11

TTLL5

TTN

TTYH3

TUBA3C

TUBGCP6

TULP4

TXNDC5

UBA6

UBAC2

UBE2K

UBL3

UBR1

UBXN2A

UBXN2B

UHRF1BP1

UHRF2

UIMC1

UQCC

USP7

UTP14A

UTP18

UTRN

VCL

VEGFA

VEGFC

VGLL2

VGLL4

VPRBP

VPS13B

VPS13C

VRK2

VSNL1

VTA1

WBP2NL

WDFY4

WDPCP

WDR70

WDR73

WDR75

WDR8

WFS1

WHSC1

WNK1

WNT3

WRN

WWC2

WWC3

WWOX

WWTR1

XIAP

XIRP2

XKR6

XKRX

XPO6

XYLT1

YIPF1

YME1L1

ZBTB16

ZC3H15

ZC3H7A

ZCCHC6

ZCWPW1

ZEB2

ZFAND3

ZFAT

ZFYVE26

ZMPSTE24

ZMYND8

ZNF100

ZNF208

ZNF257

ZNF276

ZNF318

ZNF341

ZNF346

ZNF347

ZNF366

ZNF383

ZNF385B

ZNF43

ZNF431

ZNF462

ZNF516

ZNF569

ZNF570

ZNF585B

ZNF652

ZNF673

ZNF776

ZNF804A

ZNF83

ZNF98

ZSCAN16

ZSCAN2

ZZZ3

Table S2 Summary of type III and IV CNVs

| Locus | Coordinates (hg 19) | Size (kb) | Category^a^ | No. of cases |
| --- | --- | --- | --- | --- |
| 1p34.2 | chr1:40683823-40765760 | 82 | loss | 1 |
| 1p32.2-p31.1 | chr1:57561106-77291188 | 19730 | LOH | 1 |
| 1p21.2-p13.3 | chr1:102101223-107748276 | 5647 | LOH | 1 |
| 1p13.2-p11.2 | chr1:113925870-121346616 | 7421 | LOH | 1 |
| 1q21.1-q21.2 | chr1:144945737-147104996 | 2159 | LOH | 1 |
| 2p11.2-p11.1 | chr2:89319919-91815738 | 2496 | gain | 1 |
| 2q37.3 | chr2:238552639-239318606 | 766 | gain | 1 |
| 2q37.3 | chr2:241974674-241976789 | 2 | gain | 1 |
| 2p11.2-p11.1 | chr2: 89964896-91687089 | 1722 | loss | 1 |
| 3q28 | chr3:189494234-189497803 | 4 | gain | 72 |
| 3p24.1 | chr3:30673909-30681689 | 8 | loss | 1 |
| 3q23 | chr3:141267929-141283035 | 15 | loss | 1 |
| 4q12 | chr4:55093859-55100438 | 7 | gain | 1 |
| 4p14 | chr4:39568792-39734814 | 166 | loss | 1 |
| 4p14 | chr4:39911292-39963438 | 52 | loss | 1 |
| 4p13-p11 | chr4:41460274-48907122 | 7447 | LOH | 1 |
| 4p13-p11 | chr4:41528563-48907122 | 7379 | LOH | 1 |
| 4q12 | chr4:52780048-53880619 | 1101 | LOH | 1 |
| 4q12 | chr4:52780048-54574274 | 1794 | LOH | 1 |
| 5p12-p11 | chr5:45362306-46279735 | 917 | gain | 1 |
| 5p15.2 | chr5:11053726-11063246 | 10 | loss | 1 |
| 5q31.2 | chr5:137463337-137875920 | 413 | loss | 1 |
| 6p21.31 | chr6:34618580-34728303 | 110 | loss | 1 |
| 6q13 | chr6:74059539-74341740 | 282 | loss | 1 |
| 6q24.1 | chr6:142638687-142640982 | 2 | loss | 1 |
| 6q26 | chr6:163420055-163520755 | 101 | loss | 1 |
| 6p24.3-p23 | chr6:8580437-13907710 | 5327 | LOH | 1 |
| 6p22.3-p21.33 | chr6:23833447-30685420 | 6852 | LOH | 1 |
| 6q14.3-q16.1 | chr6:86115244-93716875 | 7602 | LOH | 1 |
| 6q21-q22.31 | chr6:105572808-122014142 | 16441 | LOH | 1 |
| 7p21.3 | chr7:12193099-13429551 | 1236 | gain | 1 |
| 7p21.1 | chr7:20749353-20752852 | 4 | gain | 49 |
| 7p15.3 | chr7:23544267-23564825 | 21 | loss | 3 |
| 7p15.3 | chr7:23544267-23567813 | 24 | loss | 1 |
| 7q22.1 | chr7:98919363-99090818 | 171 | loss | 1 |
| 8p12 | chr8:30536820-30610377 | 74 | loss | 1 |
| 8q21.13 | chr8:83229772-83449931 | 220 | loss | 1 |
| 8q21.11-q21.13 | chr8:76221468-82471938 | 6250 | LOH | 1 |
| 9p13.1-p12 | chr9:39465038-41897631 | 2432 | gain | 1 |
| 9q31.2 | chr9:110066126-110093878 | 28 | gain | 1 |
| 9q33.3-q34.11 | chr9:129499616-132081244 | 2582 | loss | 1 |
| 9q34.11 | chr9:130589553-132043687 | 1454 | loss | 1 |
| 9q22.31-q22.33 | chr9:94405487-101585831 | 7180 | LOH | 1 |
| 11p15.4 | chr11:9200052-9530022 | 330 | loss | 1 |
| 11q23.3 | chr11:118624365-119086001 | 462 | loss | 1 |
| 12q24.31 | chr12:123656543-123708337 | 52 | loss | 1 |
| 12p13.1-p12.2 | chr12:12972903-20076620 | 7104 | LOH | 1 |
| 12p11.21-p11.1 | chr12:32825906-34252870 | 1427 | LOH | 1 |
| 12q11-q13.11 | chr12:37864924-46465516 | 8601 | LOH | 1 |
| 12q14.1-q15 | chr12:62268292-68586033 | 6318 | LOH | 1 |
| 14q12 | chr14:24629384-24704536 | 75 | loss | 1 |
| 14q12 | chr14:24677440-24680914 | 3 | loss | 1 |
| 14q23.3-q24.3 | chr14:66475085-78362176 | 11887 | LOH | 1 |
| 15q15.1 | chr15:41610817-41739757 | 129 | loss | 1 |
| 17q12 | chr17:36919637-37572368 | 653 | loss | 1 |
| 17q21.32-q21.33 | chr17:47304444-47490707 | 186 | loss | 1 |
| 17q21.32-q21.33 | chr17:47374194-47492451 | 118 | loss | 1 |
| 17q21.33 | chr17:49126263-49275529 | 149 | loss | 1 |
| 17q21.32-q22 | chr17:44992844-52384726 | 7392 | LOH | 1 |
| 17q21.33-q22 | chr17:48359279-53889470 | 5530 | LOH | 1 |
| 17q22-q24.2 | chr17:54966805-65072775 | 10106 | LOH | 1 |
| 18q22.3 | chr18:70427261-70433706 | 6 | gain | 1 |
| 18q22.3 | chr18:70533867-70611412 | 78 | gain | 1 |
| 18q11.2 | chr18:20751522-20755362 | 4 | loss | 1 |
| 18q12.2-q12.3 | chr18:33975988-39401882 | 5426 | LOH | 1 |
| 19p13.3-p13.2 | chr19:6359738-7211068 | 851 | loss | 1 |
| 19p13.2 | chr19:8567368-8654857 | 87 | loss | 1 |
| 19p13.11 | chr19:17213187-18288374 | 1075 | loss | 1 |
| 20p13 | chr20:2798690-3005149 | 206 | loss | 1 |
| 20q11.22-q11.23 | chr20:33975608-34497401 | 522 | loss | 1 |
| 21q22.11 | chr21:34696488-34699955 | 3 | loss | 1 |
| 22q11.21 | chr22:18729944-19174936 | 445 | loss | 1 |
| Xq21.33 | chrX:96771368-96831965 | 61 | gain | 1 |
| Xq26.2 | chrX:130964076-131697519 | 733 | gain | 1 |
| Xq27.1-q27.2 | chrX:139739465-140414348 | 675 | gain | 1 |
| Xp11.4-p11.21 | chrX:41945535-57999932 | 16054 | LOH | 1 |
| Xp11.3-p11.21 | chrX:43595678-57999932 | 14404 | LOH | 1 |
| Xp11.23-p11.21 | chrX:46791335-57999932 | 11209 | LOH | 1 |
| Xp11.23-p11.21 | chrX:48467186-57999932 | 9533 | LOH | 1 |
| Xp11.23-p11.21 | chrX:49504886-57999932 | 8495 | LOH | 1 |
| Xp11.22-p11.21 | chrX:50357704-57999932 | 7642 | LOH | 1 |
| Xq11.2-q13.1 | chrX:63663978-66956221 | 3292 | LOH | 1 |
| Xq11.2-q12 | chrX:63663978-67370962 | 3707 | LOH | 1 |
| Xq11.2-q13.1 | chrX:63663978-68754737 | 5091 | LOH | 1 |
| Xq11.2-q13.1 | chrX:63663978-69423721 | 5760 | LOH | 1 |
| Xq11.2-q13.1 | chrX:63663978-69489530 | 5826 | LOH | 1 |
| Xq11.2-q13.1 | chrX:63663978-69579377 | 5915 | LOH | 1 |
| Xq22.3-q23 | chrX:105466607-114260915 | 8794 | LOH | 1 |
| Xq27.3-q28 | chrX:144910142-151090892 | 6181 | LOH | 1 |
| Xq27.3-q28 | chrX:145280008-152492059 | 7212 | LOH | 1 |
| Yq11.223-q11.23 | chrY:24366701-28478618 | 4112 | gain | 1 |
| Yq11.223 | chrY:24636946-25383954 | 747 | gain | 1 |
| Yq11.223-q11.23 | chrY:24874263-28071741 | 3197 | gain | 1 |

^a^The category of detected copy number variations (CNVs) comprised gain (duplication) and loss (deletion). LOH, loss of heterozygosity.

Table S3 Primers of qPCR

| Patient | Primer | Sequence^a^ |
| --- | --- | --- |
| 1 | TBX1-F | CATGGCCCAGAATGACTACTCAG |
|  | TBX1-R | GTATGGGGTGCCTCATGTCC |
|  | SERPIND1-F | ATCCTTGCACACCTGACTCTG |
|  | SERPIND1-R | CAAGAATGGTGCTAGTTGTGTACC |
| 2 | SCARF2-F | AGCTTTCGCTTGGGAGGAAG |
|  | SCARF2-R | CGCTGTCATCCTTACCTACGAC |
|  | SERPIND1-F | ATCCTTGCACACCTGACTCTG |
|  | SERPIND1-R | CAAGAATGGTGCTAGTTGTGTACC |
| 3 | SHOX-F | GCATCCGATAGAAGCTGTTGC |
|  | SHOX-R | TCACCGTGGTCTCATAACTTTCG |
|  | RPS4Y1-F | TCAGCATCGAGAAGACAGGTG |
|  | RPS4Y1-R | TCACTTGACTTGTGCAACATACC |
| 4 | POLR2B-F | CCTAGAAACACATACCAGTCTGC |
|  | POLR2B-R | AGACCGTGTAGTCACAAGTGG |
|  | PDGFRA-F | CAGATGTAGCCTTTGTACCTCTAGG |
|  | PDGFRA-R | GGATCAGTTGTGCGACAAGG |
| 5 | PDGFRA-F | CAGATGTAGCCTTTGTACCTCTAGG |
|  | PDGFRA-R | GGATCAGTTGTGCGACAAGG |
|  | CHIC2-F | GAGACCCAATGTAGTATGGAACACC |
|  | CHIC2-R | TGGAGGAGCAAACATGCATACA |
| VUS  proband | RASA2-F | TTCAGGATCGACTTGTGGAACA |
|  | RASA2-R | CCTTACCAGGCTTGATGAGAGG |

^a^The direction of primer sequence is from 5’ to 3’. VUS, variants of uncertain significance.

A

Patient 1, deletion, 22q11.21, chr22:18729944-21704972, 3Mb


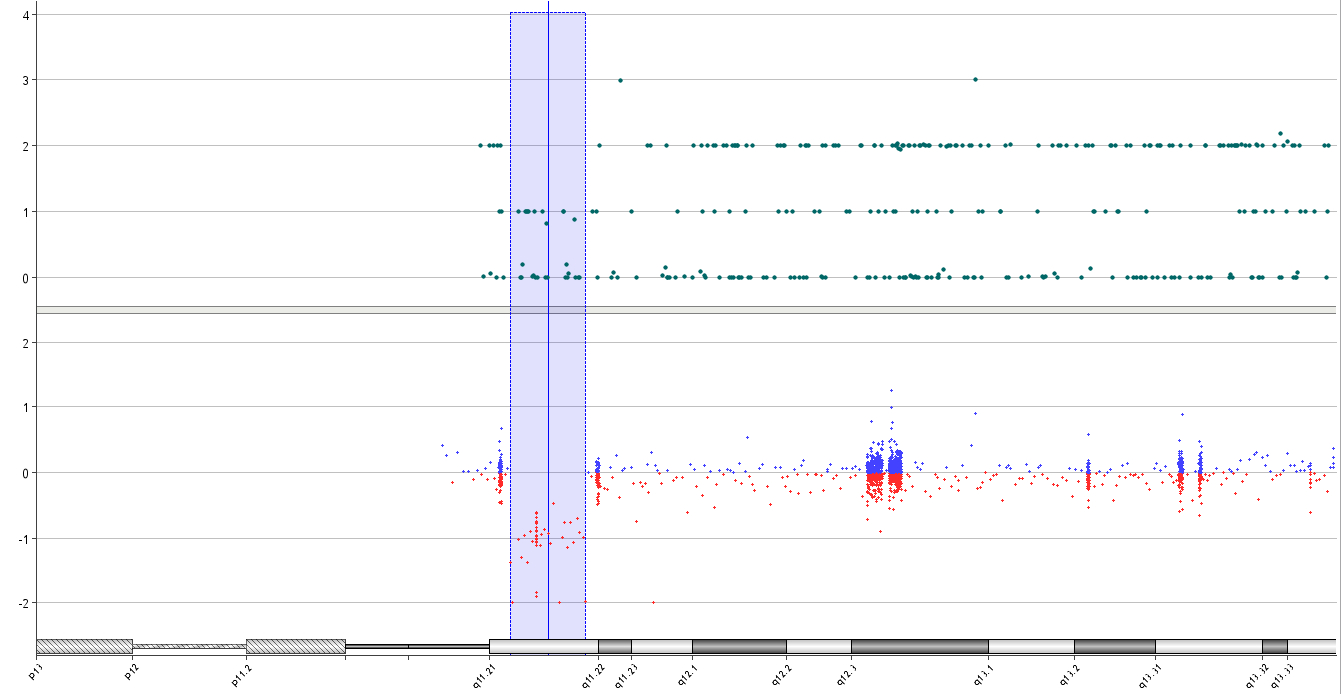


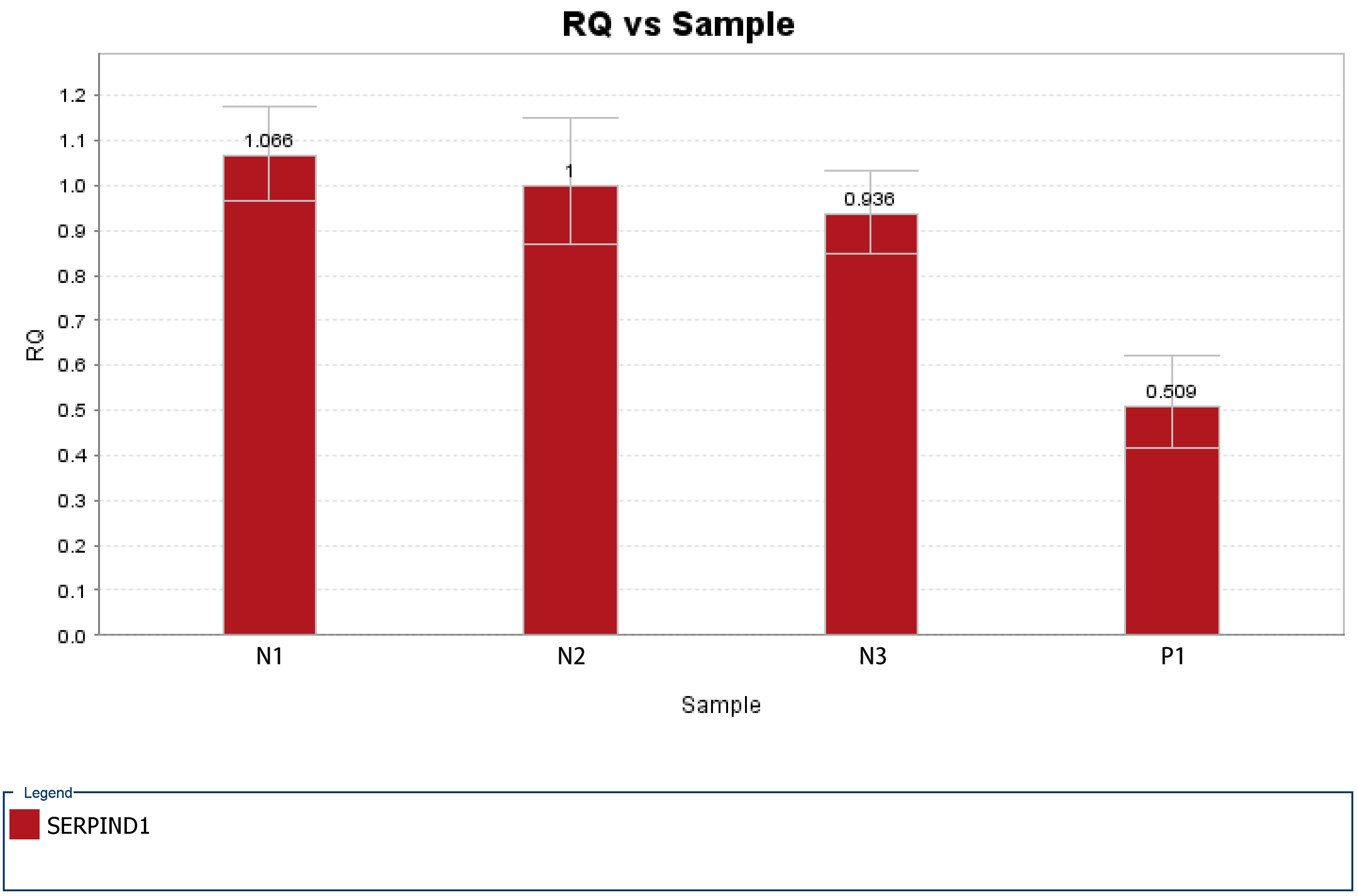

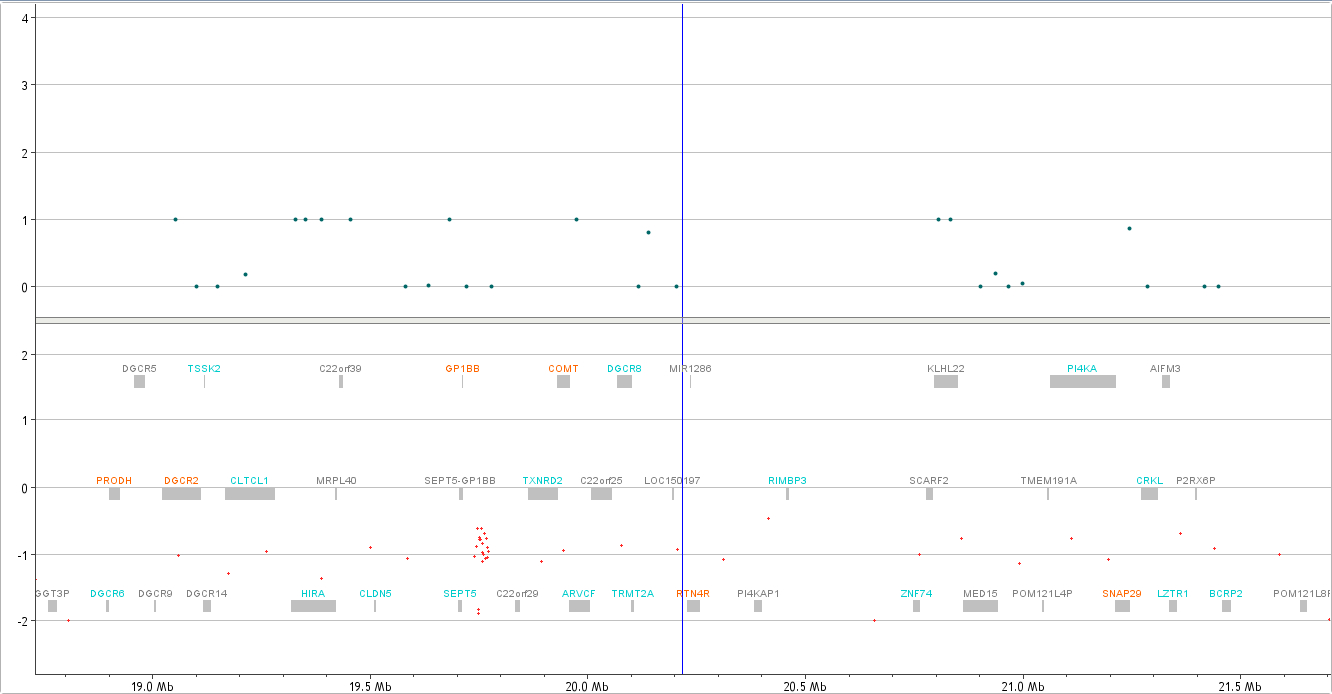


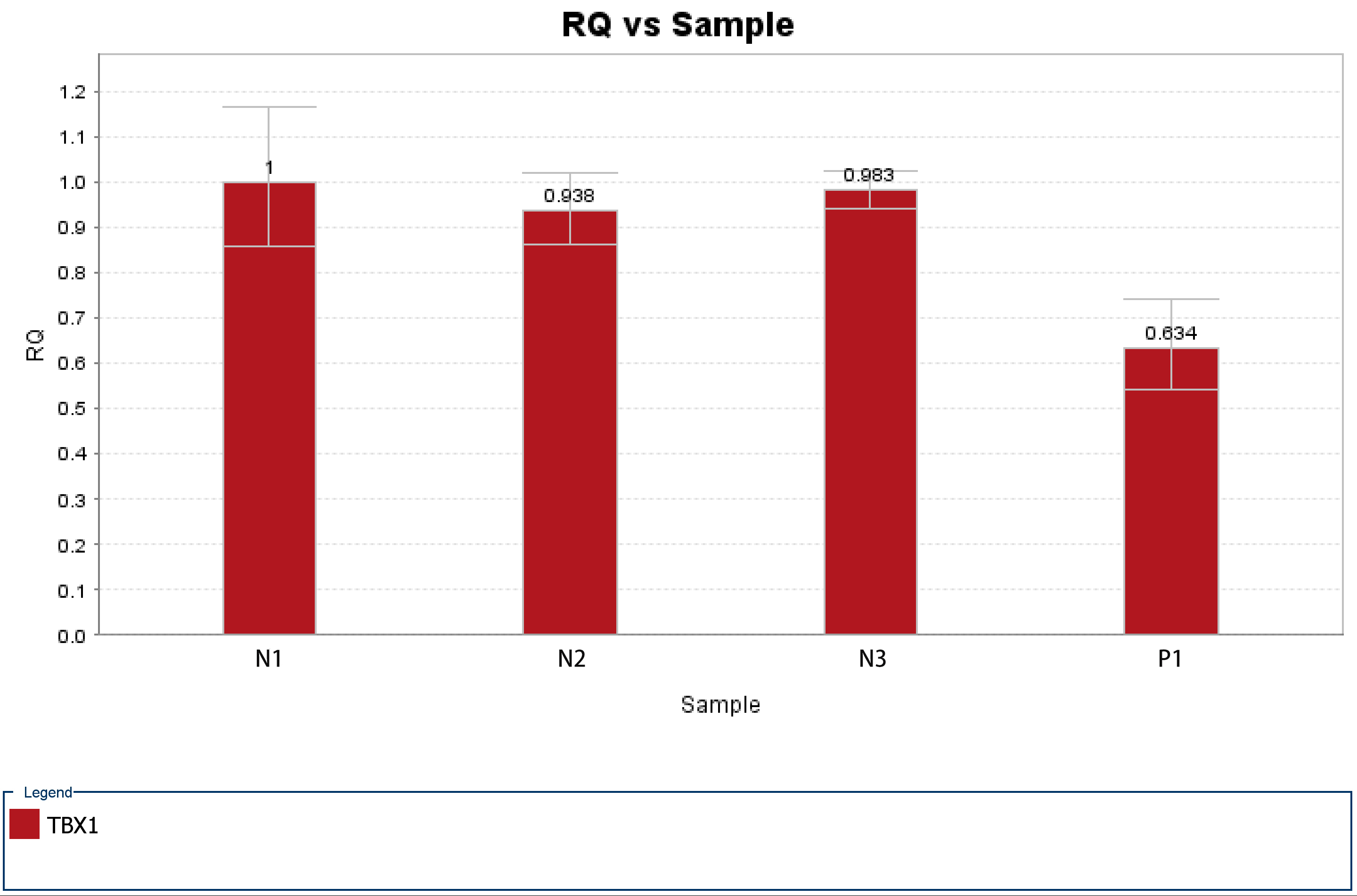


B

Patient 2, deletion, 22q11.21, chr22:20659547-21704972, 1Mb


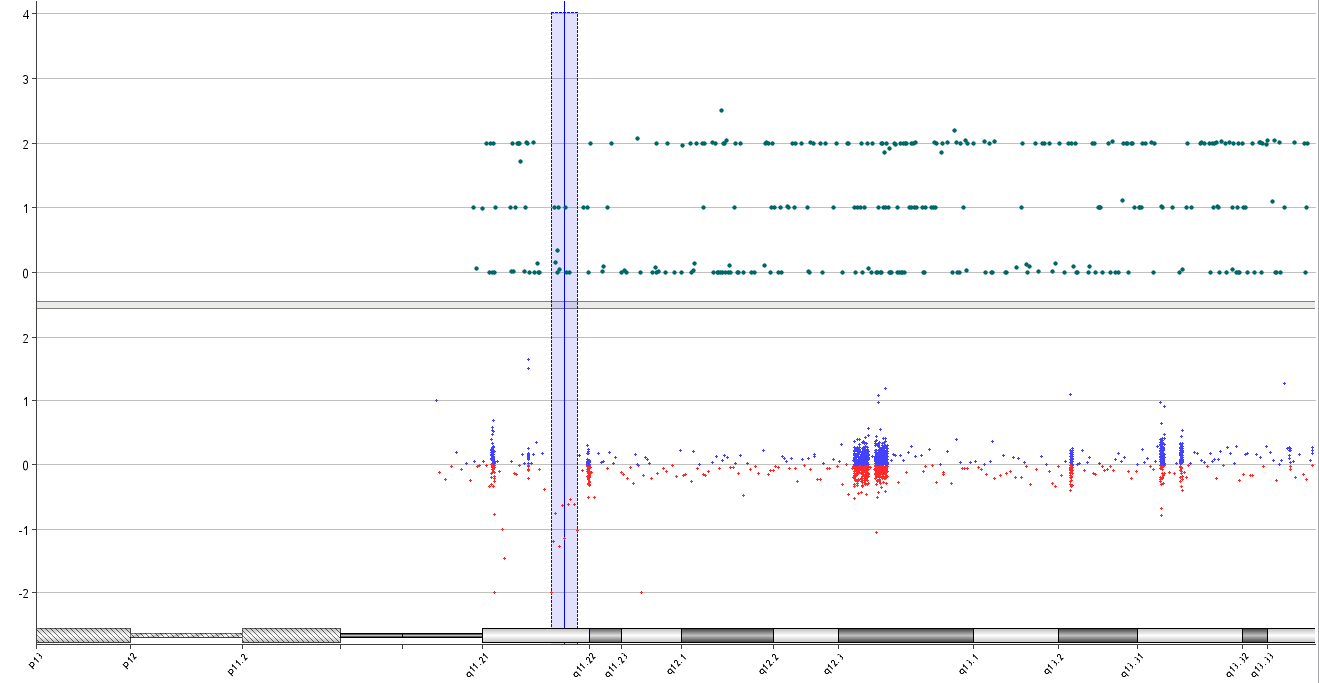


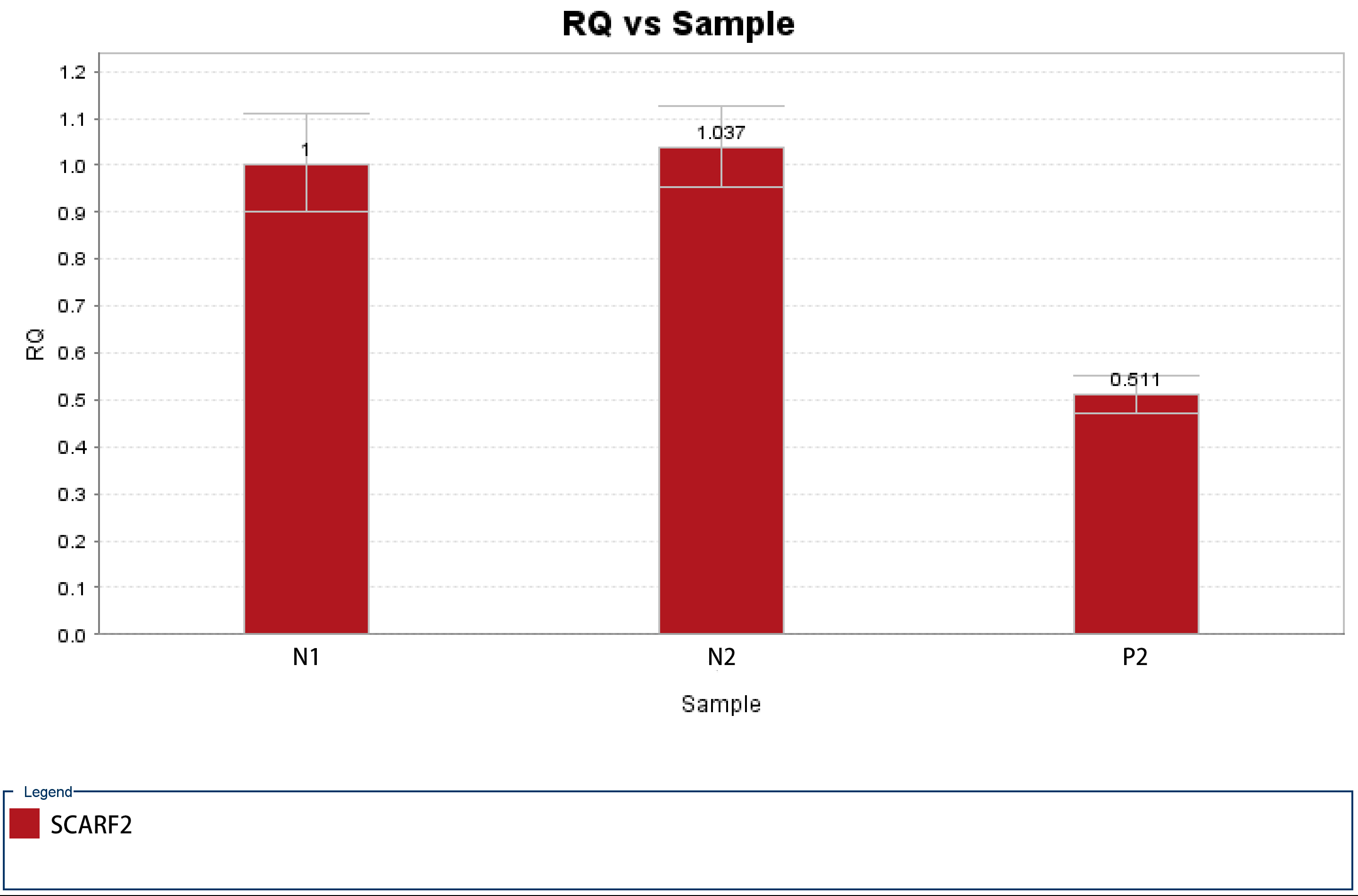

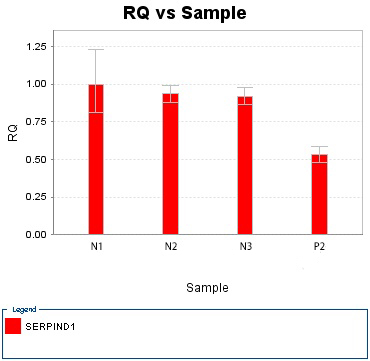

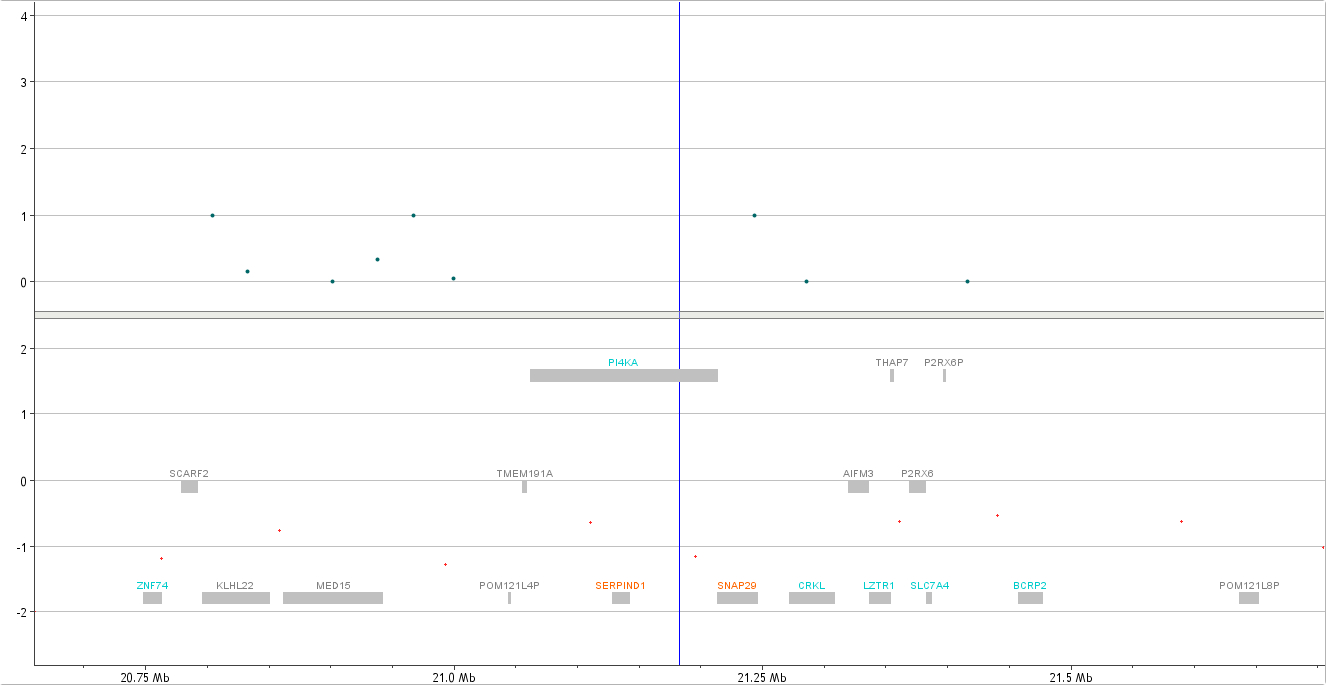


C

Patient 3, duplication, Yp11.32-p11.2, chrY:545173-10045809, 9.5Mb


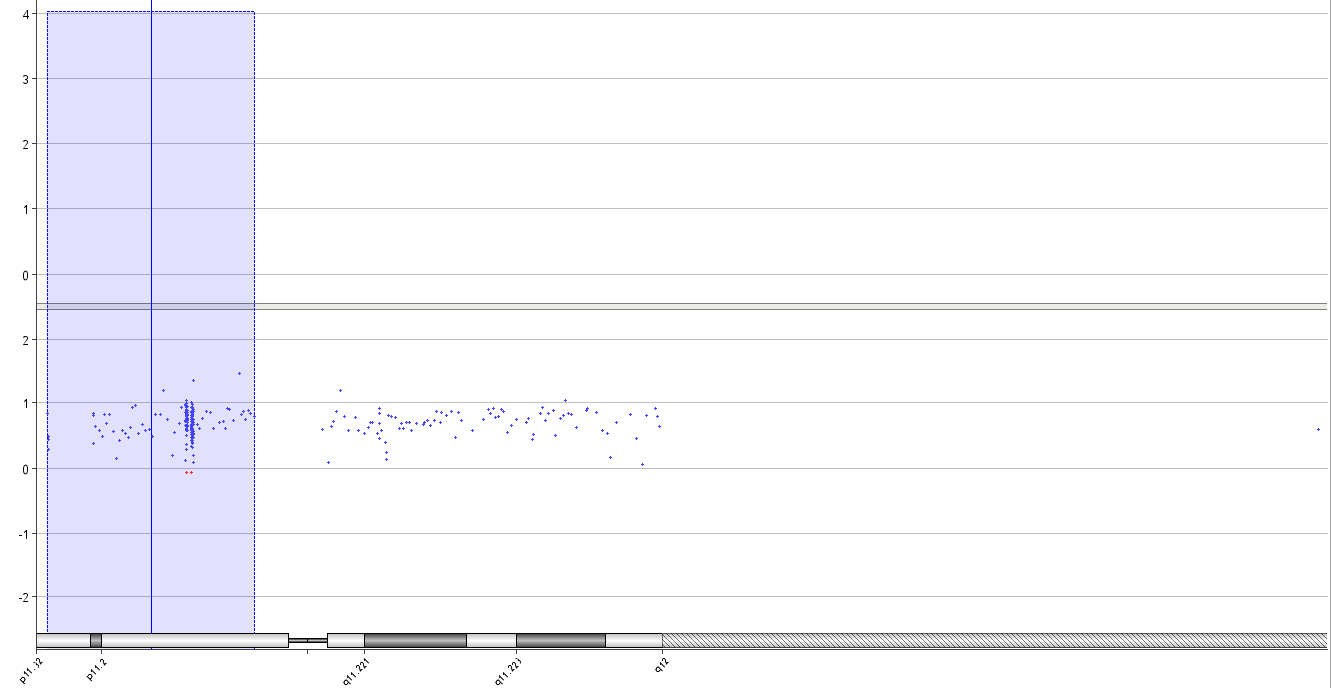


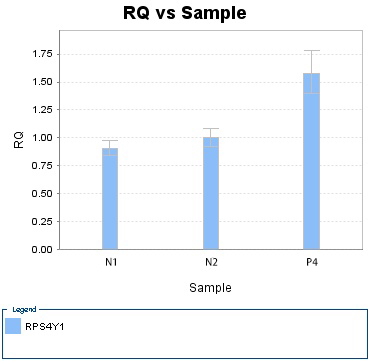

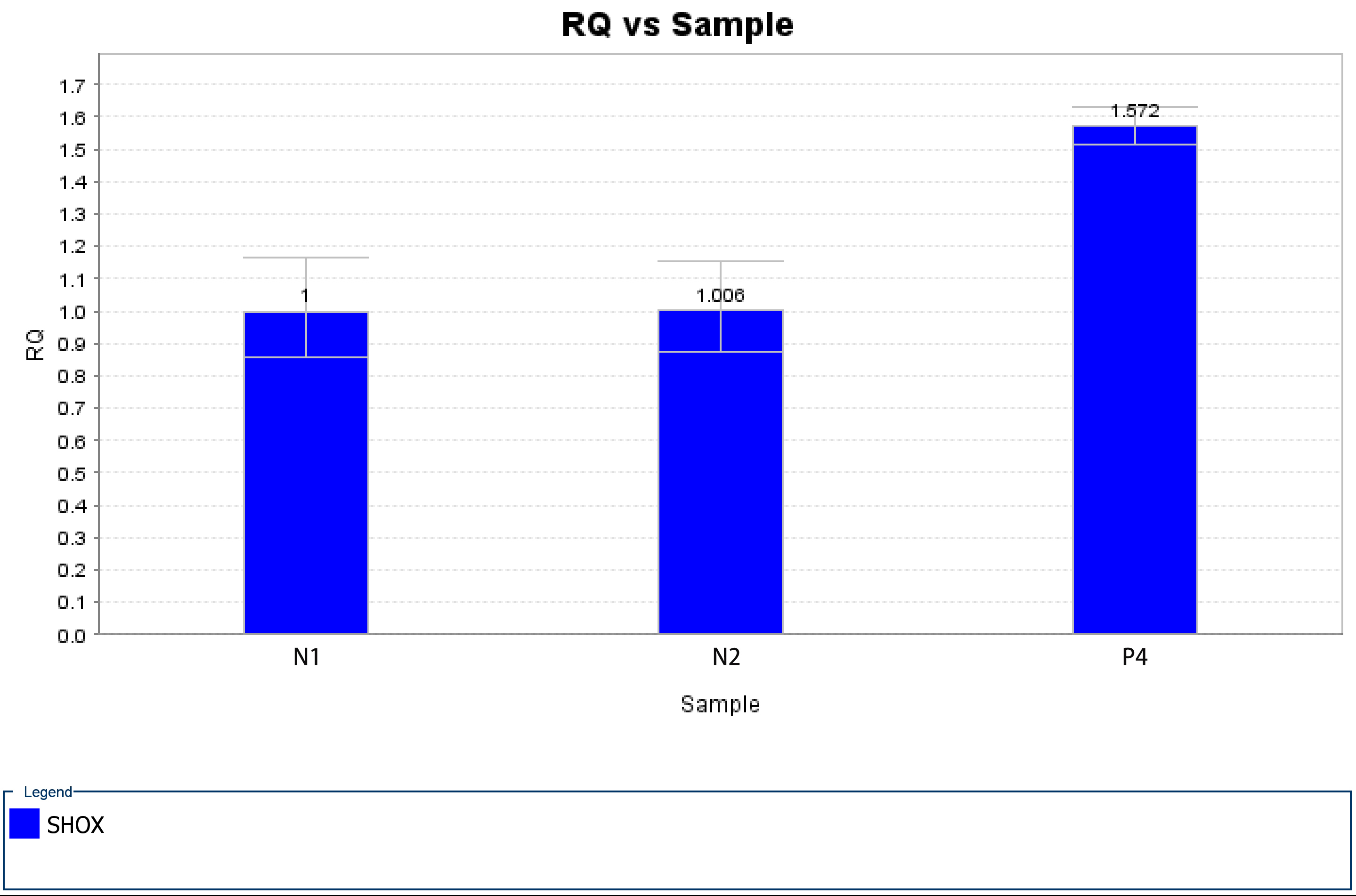

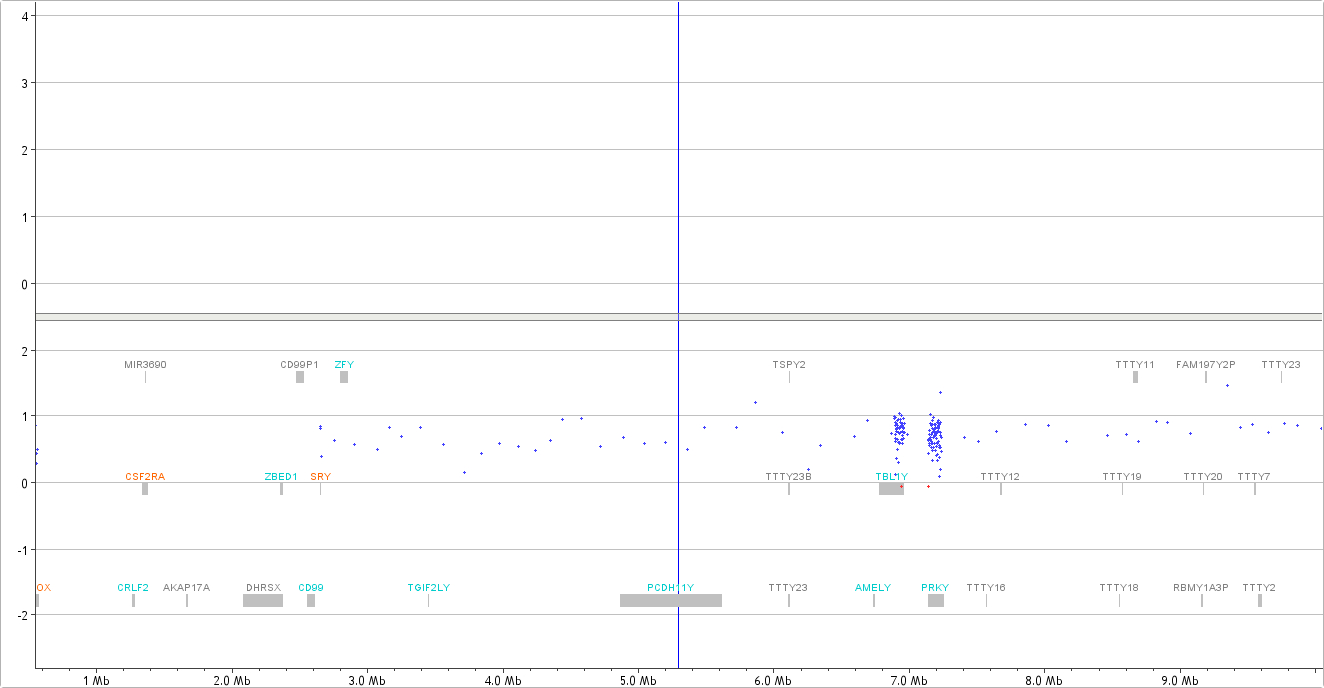


D

Patient 4, duplication, 4q11-q13.1, chr4:52697788-59679060, 7Mb


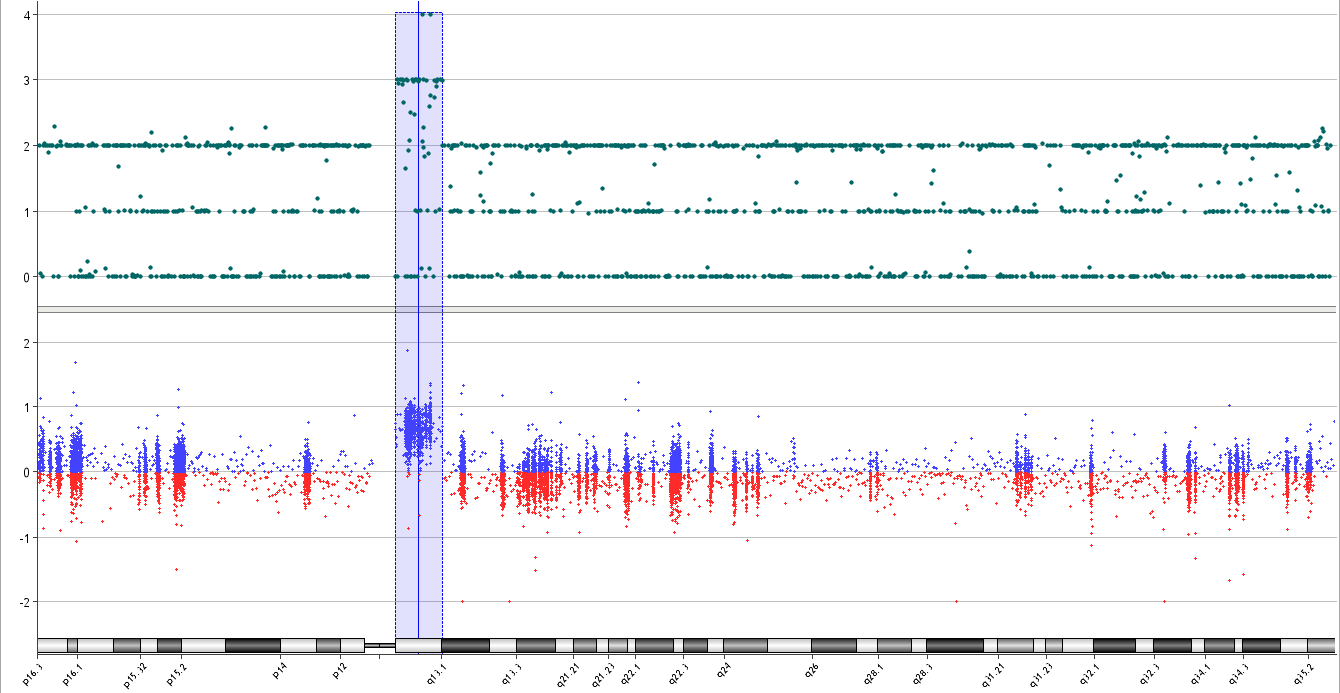


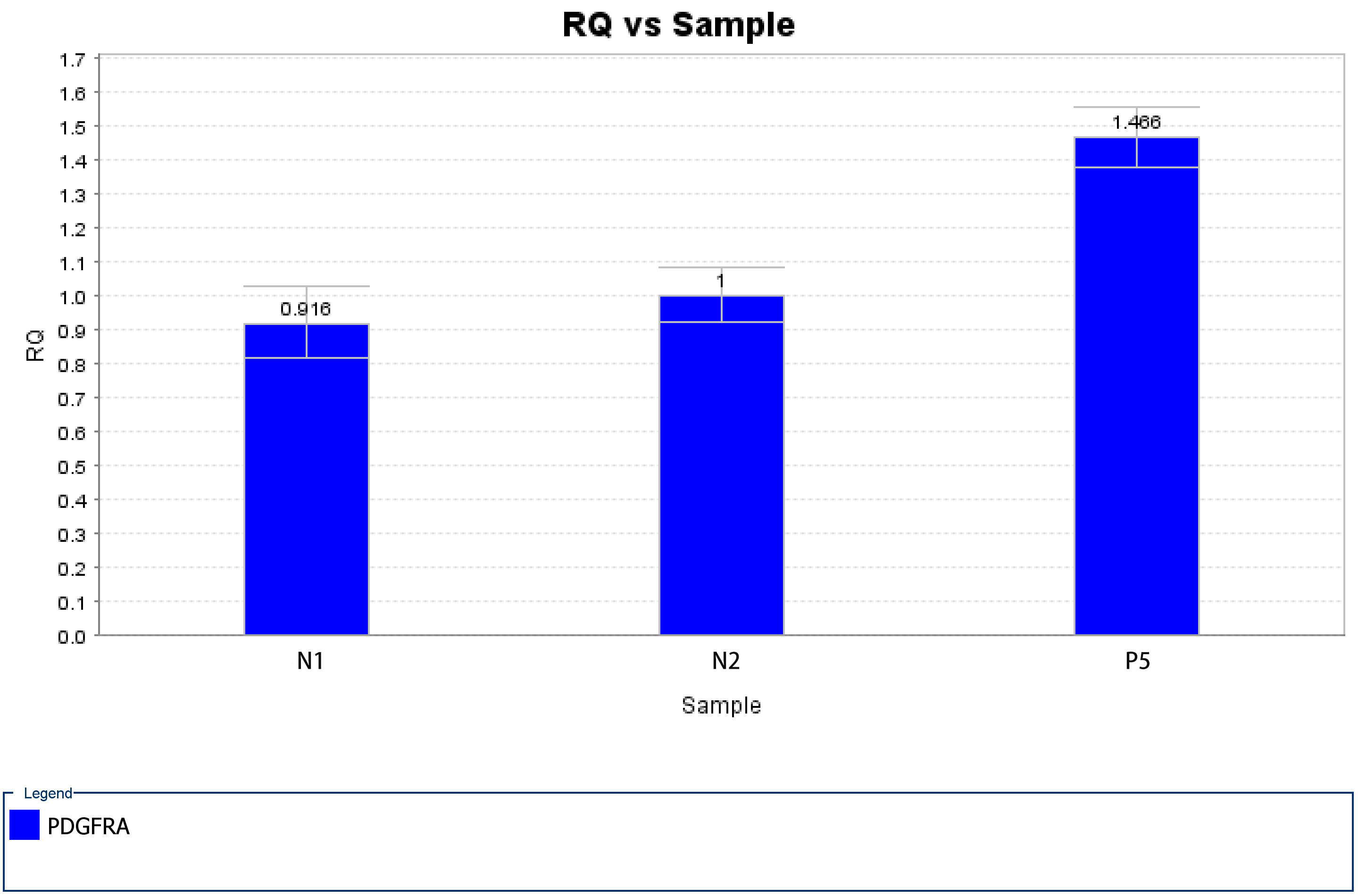

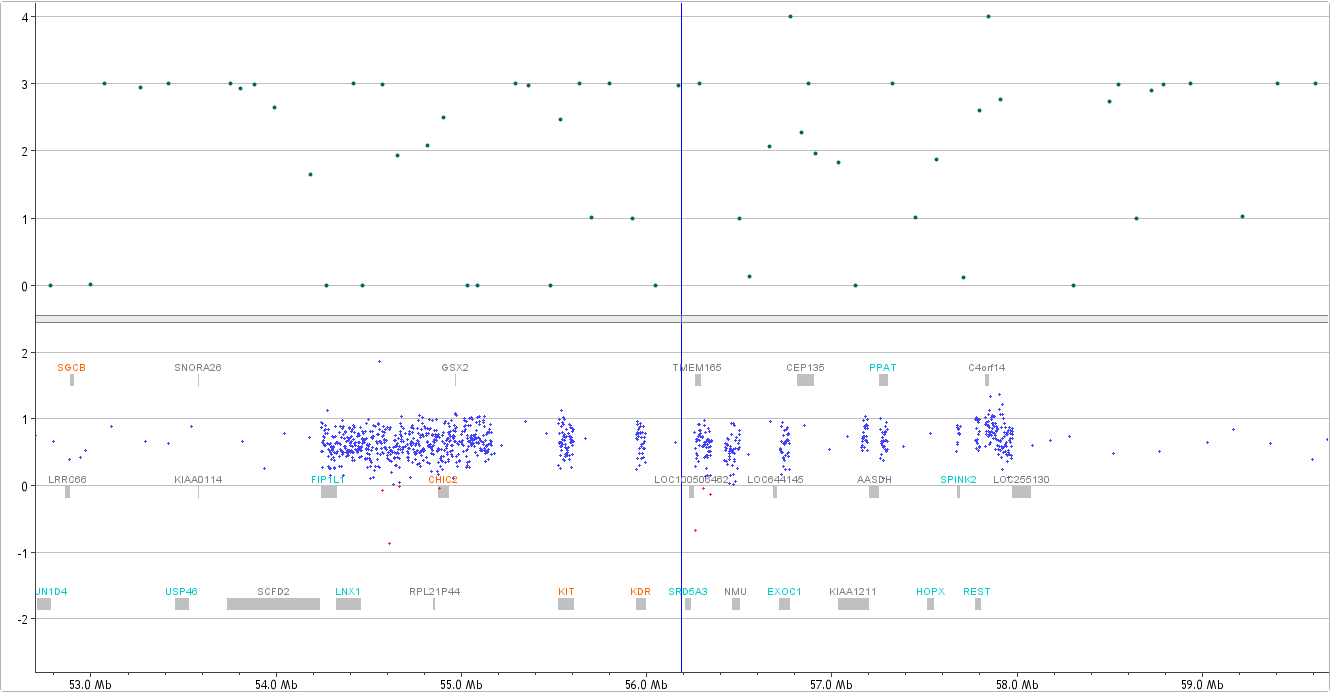


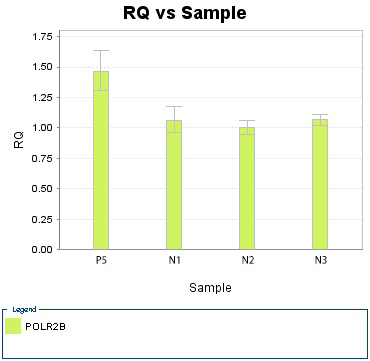


E

Patient 5, duplication, 4q12, chr4:54749407-55157199, 0.4Mb


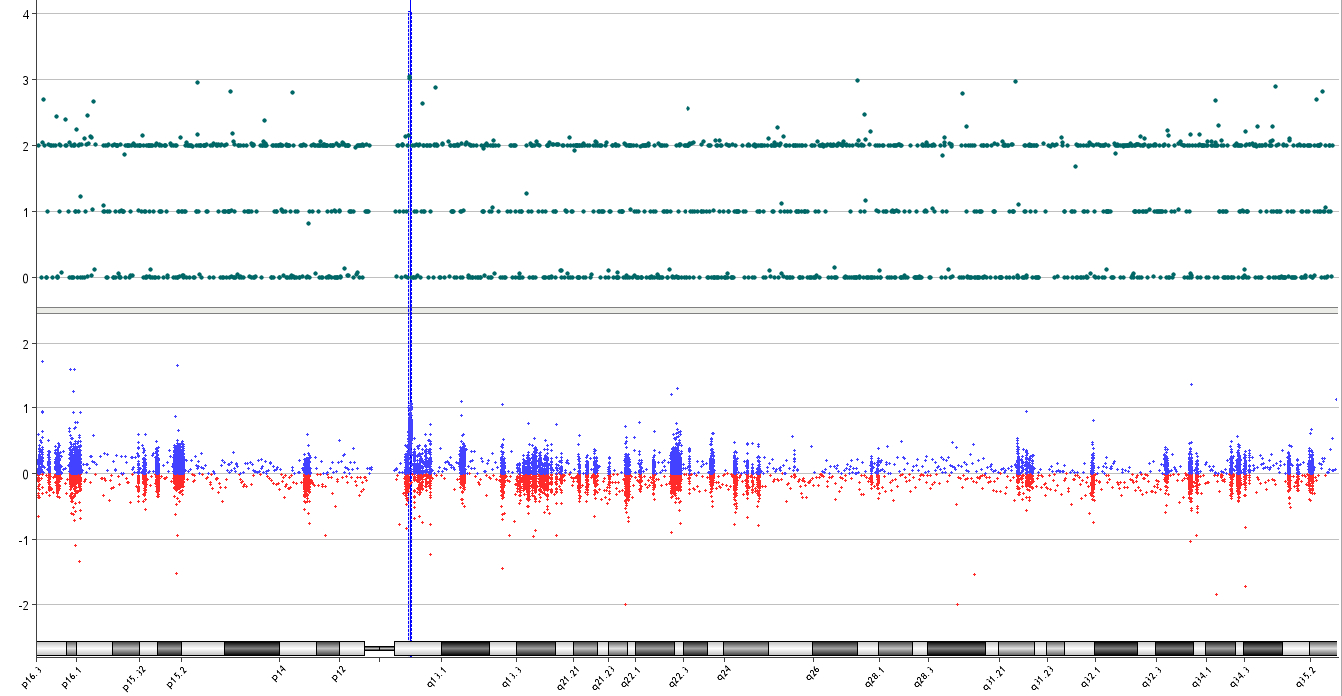


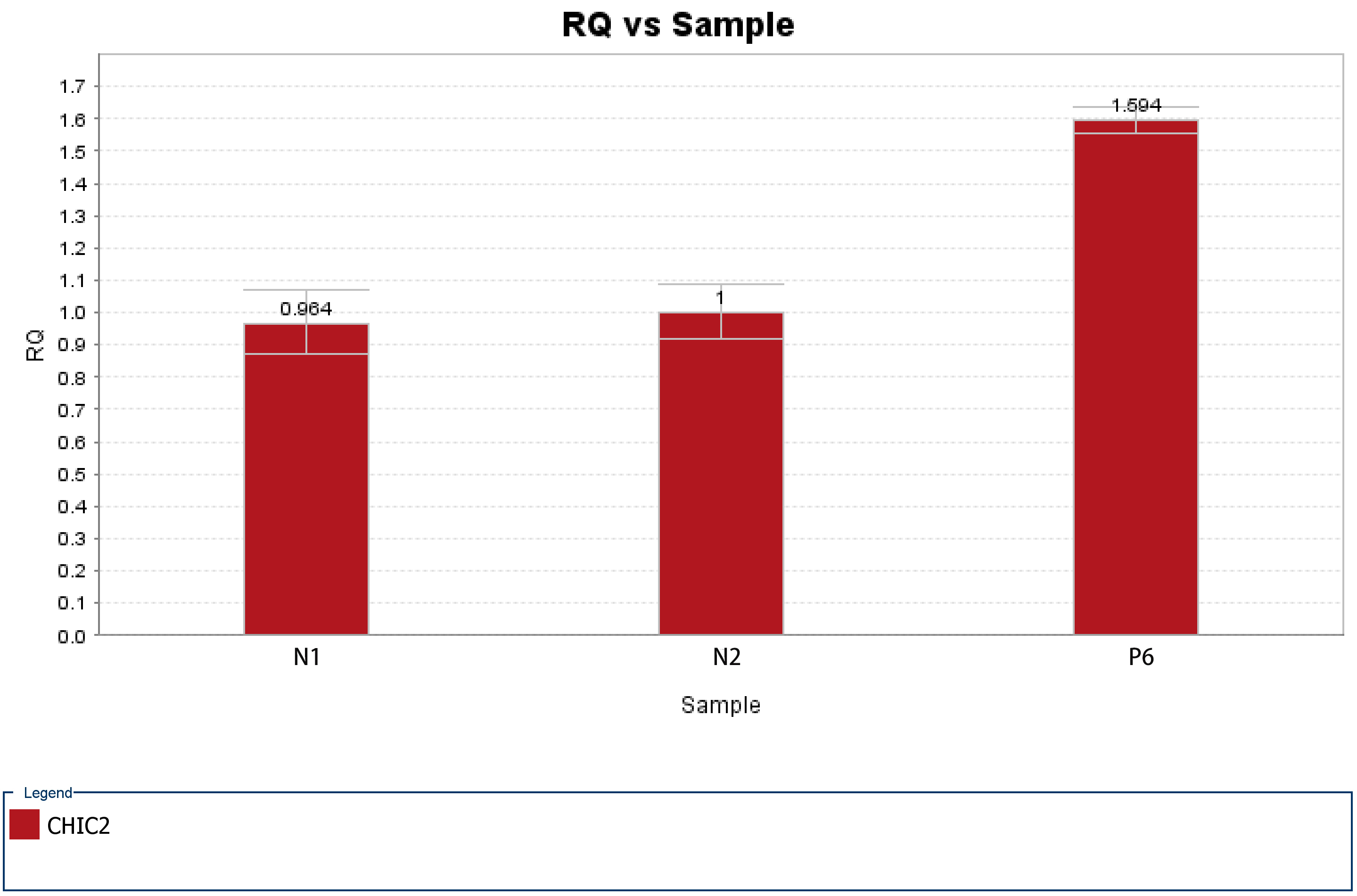

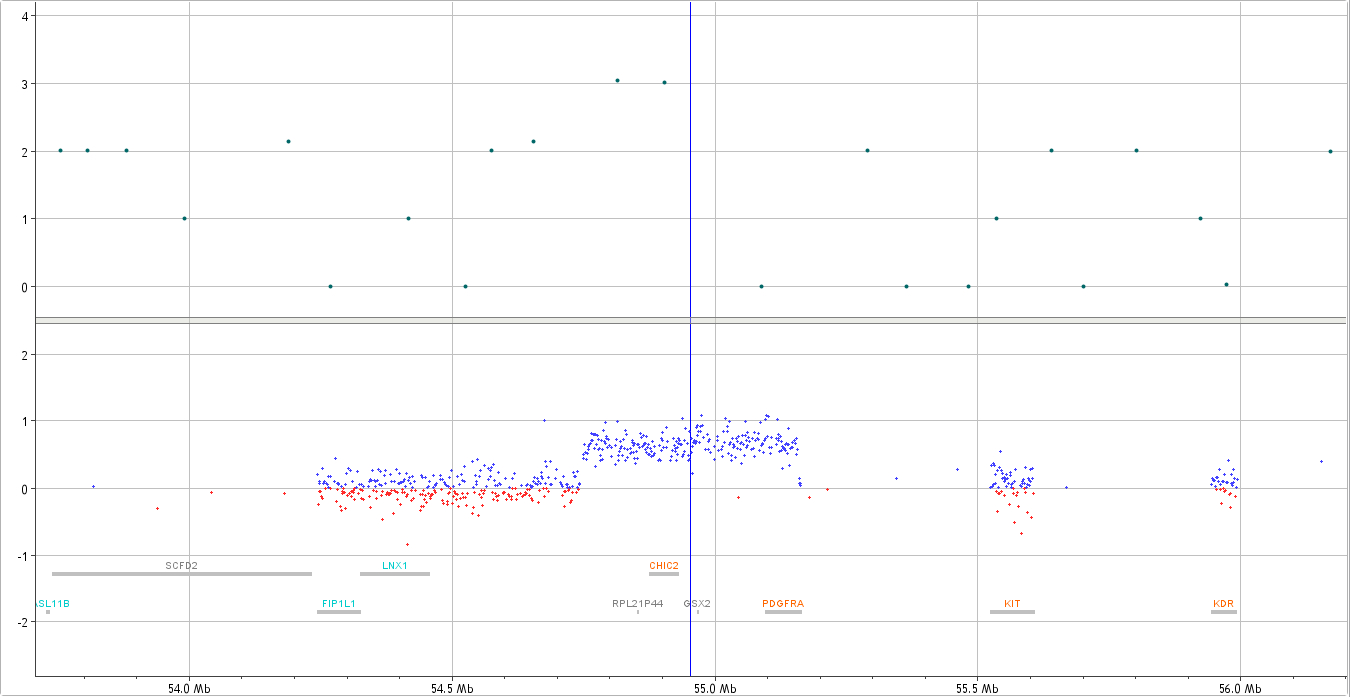


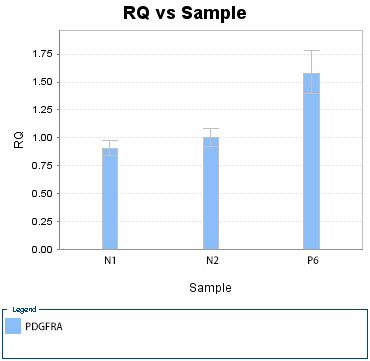


F

VUS proband, deletion, 3q23, chr3:141267929-141283035, 15kb


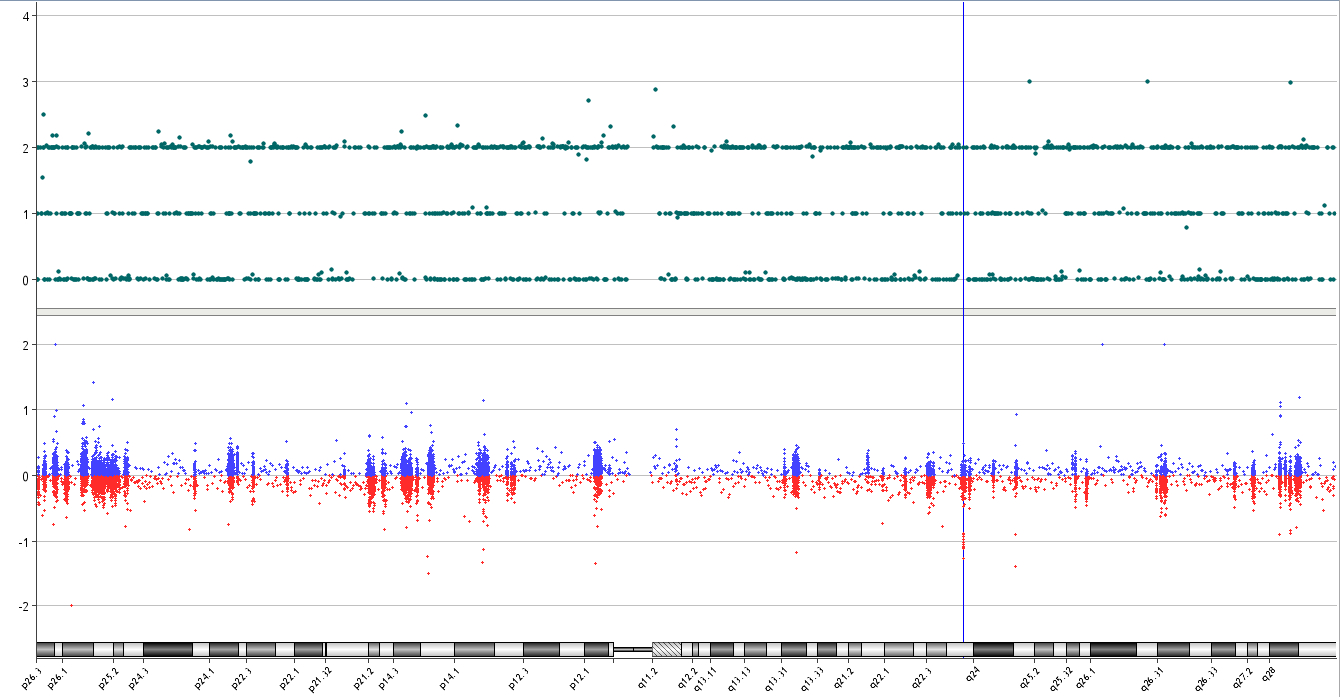


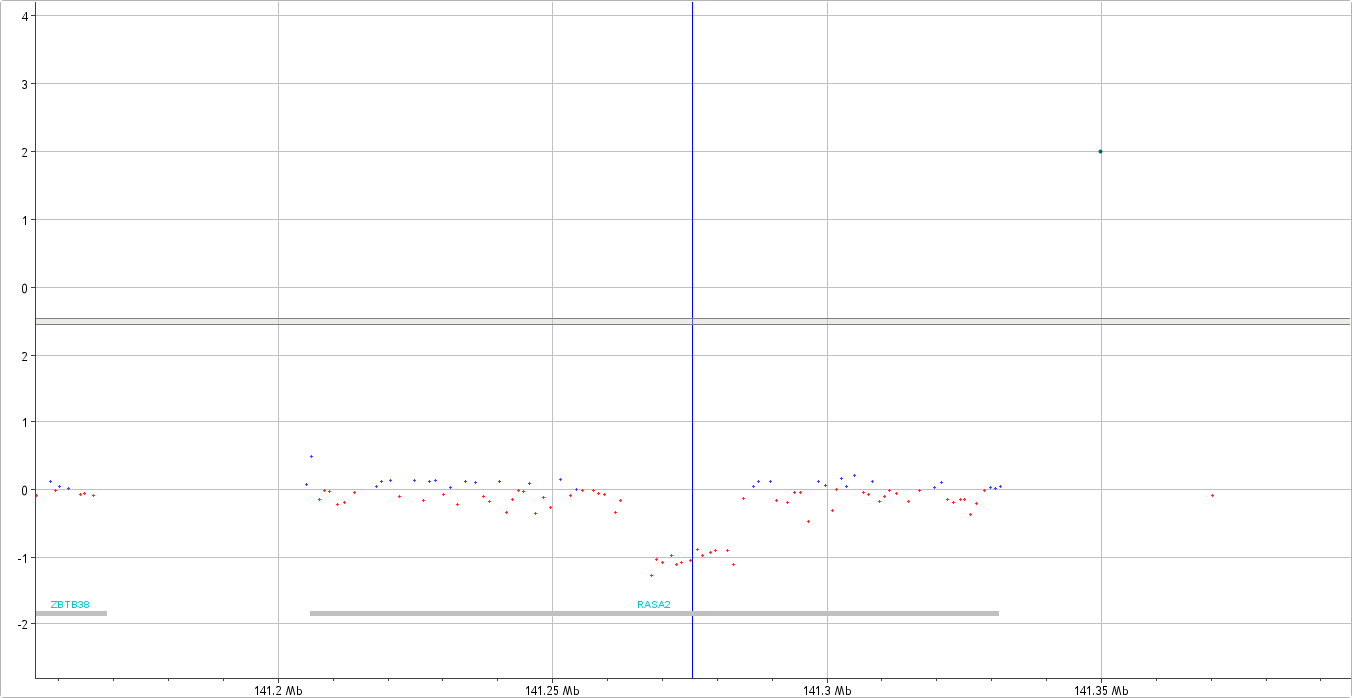


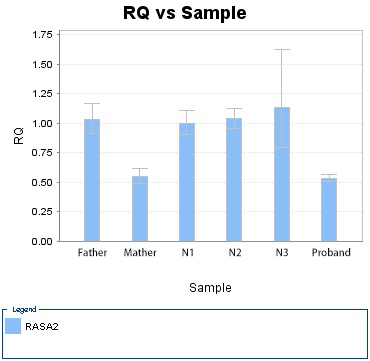


Fig. S1 Screenshots of chromosome microarray and qPCR. From A to F, they were patient 1 to 5 and a boy categorized into variants of uncertain significance. N1 to 3 were normal controls.
